# Sperm Functional Genome Associated With Bull Fertility

**DOI:** 10.3389/fvets.2021.610888

**Published:** 2021-06-22

**Authors:** Memmet Özbek, Mustafa Hitit, Abdullah Kaya, Frank Dean Jousan, Erdogan Memili

**Affiliations:** ^1^Department of Histology and Embryology, Faculty of Veterinary Medicine, Burdur Mehmet Akif Ersoy University, Burdur, Turkey; ^2^Department of Genetics, Faculty of Veterinary Medicine, Kastamonu University, Kastamonu, Turkey; ^3^Department of Artificial Insemination and Reproduction, Faculty of Veterinary Medicine, Selcuk University, Konya, Turkey; ^4^Department of Animal and Dairy Sciences, Mississippi State University, Starkville, MS, United States

**Keywords:** proteomics, metabolomics, transcriptomics, DNA methylome, chromatin dynamics, bull fertility

## Abstract

Bull fertility is an important economic trait in sustainable cattle production, as infertile or subfertile bulls give rise to large economic losses. Current methods to assess bull fertility are tedious and not totally accurate. The massive collection of functional data analyses, including genomics, proteomics, metabolomics, transcriptomics, and epigenomics, helps researchers generate extensive knowledge to better understand the unraveling physiological mechanisms underlying subpar male fertility. This review focuses on the sperm phenomes of the functional genome and epigenome that are associated with bull fertility. Findings from multiple sources were integrated to generate new knowledge that is transferable to applied andrology. Diverse methods encompassing analyses of molecular and cellular dynamics in the fertility-associated molecules and conventional sperm parameters can be considered an effective approach to determine bull fertility for efficient and sustainable cattle production. In addition to gene expression information, we also provide methodological information, which is important for the rigor and reliability of the studies. Fertility is a complex trait influenced by several factors and has low heritability, although heritability of scrotal circumference is high and that it is a known fertility maker. There is a need for new knowledge on the expression levels and functions of sperm RNA, proteins, and metabolites. The new knowledge can shed light on additional fertility markers that can be used in combination with scrotal circumference to predict the fertility of breeding bulls. This review provides a comprehensive review of sperm functional characteristics or phenotypes associated with bull fertility.

## Introduction

Projections indicate that the world population will rise to 9 billion people by 2,050, requiring a 50% increase in food production (1) to satisfy the demands of a growing population. Animal agriculture will benefit from technological advances to produce livestock and their byproducts more efficiently and economically. Biotechnology is crucial in promoting sustainability of livestock production in order to meet these demands for high-quality food products with less environmental impact. Important advances in livestock production have been achieved through reproductive biotechnology (2). Bull fertility, defined as the ability of a spermatozoa to fertilize an oocyte and support embryonic development (3), and accurate evaluation of semen quality parameters used as predictors of bull fertility, remain as important research imperatives to further enhance improvements in genetic selection in cattle (4). Generally, in studies, bull fertility is calculated based on the conception rate. For each bull, at least 100 insemination records are considered reliable data to evaluate the correct conception rate. Pregnancy diagnosis is controlled by trans-rectal ultrasonographic examination within 45–50 days following artificial insemination. The conception rate of each bull was plotted in a graph, and the standard deviation (SD) and the mean were calculated. The criterion for selecting high-fertile bulls was conception rate more than “mean + 1 or 2 SD,” while those below “mean – 1 or 2 SD” were considered as low-fertile bulls (5, 6).

Bulls are evaluated based on a breeding soundness exam (BSE) that is composed of an inspection of semen characteristics (phenotypes) combined with phenotypic features. Despite great efforts put into evaluating bulls using BSE, bull fertility is deemed suboptimal under field conditions, with a conception rate varying from 20 to 40% (7). Such differences may be due to the presence of subtle sperm abnormalities that might not be determined using current, established techniques. Semen evaluation tests, such as abnormalities, concentration, volume, membrane integrity, and motility, are now being conducted to predict the quality of semen samples for cryopreservation and subsequent use for artificial insemination. Although the standard semen evaluation procedures can help visually recognize poor-quality sperm, they are not enough to detect potential markers of subfertile bulls (7, 8). Since spermatogenesis in the bovine bull takes 61 days from spermatogonia to fully matured spermatozoa (9), there is ample time for molecular, cellular, and physiological errors to occur that can hamper sperm production and render infertility. Defects in the male germ cells during fetal life may be more probable causes of infertility than defects incurred in later phases of development, such as neonatal and postnatal periods (10). Therefore, more comprehensive studies spanning developmental stages and robust methods are needed to accurately ascertain semen quality and predict bull fertility for precision animal agriculture (11).

Genome-wide association studies (GWAS) have been effective in applying dense genetic markers, such as single-nucleotide polymorphism (SNP) markers, to determine genomic regions associated with economically important phenotype such as fertility (12). There are several studies showing a relationship between genomic regions and quantitative trait loci (QTL) and male reproductive traits in cattle (Table 1). Using a comprehensive genomic analysis on bulls, Han and Peñagaricano (25) demonstrated approximately eight genomic regions that are highly associated with bull fertility where most of these genomic regions contain genes including *Ckb, Kat8, Igf1r*, and *Tdrd9*, which are associated with sperm physiology, such as sperm motility and sperm–egg interaction. Feugang et al. (15) reported that polymorphisms in two bovine genes encoding sperm head proteins, collagen type I alpha 2 chain, and integrin subunit beta 5 are associated with bull fertility. In addition, Tüttelmann et al. (26) showed that polymorphisms in *Prm1* and *Prm2* genes were associated with human sperm quality. An SNP in *Spata1*, a gene implicated in sperm head structure, has been shown to be related to stallion fertility (27). Because the inheritance of fertility is low and is influenced by environmental and epigenetic factors, there are fewer genetic markers associated with fertility.

**Table 1:** Genomic regions and quantitative trait loci demonstrated to be associated with bovine male reproductive traits.

**Chromosome**	**Positions^**a**^ (Mb BTAU4.0)**	***N* Markers^**b**^**	**Phenotype**	***N* Animalsy^**c**^**	**Breed**	**References**
8	93 cM (MCM64−71 Mb)	263 MS	Dystocia and stillbirth	888	Holstein	(13)
5	70 cM	130	FSH serum concentration	126	MARC herd	(14)
29	44 cM	130	Age at puberty in males	126	MARC herd	(14)
29	44 cM	130	Testicular weight and volume	126	MARC herd	(14)
1	70.3 Mb	8,207 SNP	Noncompensatory fertility in bulls (semen)	221	Holstein	(15)
4	12.0 Mb	8,207 SNP	Noncompensatory fertility in bulls (semen)	221	Holstein	(15)
14	22 Mb	43,863 SNP	Paternal calving ease	1,800	German Fleckvieh	(16)
21	3.1 Mb	43,863 SNP	Paternal calving ease	1,800	German Fleckvieh	(16)
15	74.7 Mb	45,878 SNP	Daughter stillbirth	1,654	Holstein	(17)
3	30.28 Mb	38,416 SNP	Noncompensatory fertility in bulls (semen)	795	Holstein	(18)
4	17.35 Mb	38,416 SNP	Noncompensatory fertility in bulls (semen)	795	Holstein	(18)
4	76.89 Mb	38,416 SNP	Noncompensatory fertility in bulls (semen)	795	Holstein	(18)
4	109.13 Mb	38,416 SNP	Noncompensatory fertility in bulls (semen)	795	Holstein	(18)
5	47.38 Mb	38,416 SNP	Noncompensatory fertility in bulls (semen)	795	Holstein	(18)
6	39.71 Mb	38,416 SNP	Noncompensatory fertility in bulls (semen)	795	Holstein	(18)
8	89.91 Mb	38,416 SNP	Noncompensatory fertility in bulls (semen)	795	Holstein	(18)
10	81.45 Mb	38,416 SNP	Noncompensatory fertility in bulls (semen)	795	Holstein	(18)
12	29.53 Mb	38,416 SNP	Noncompensatory fertility in bulls (semen)	795	Holstein	(18)
12	45.01 Mb	38,416 SNP	Noncompensatory fertility in bulls (semen)	795	Holstein	(18)
13	36.68 Mb	38,416 SNP	Noncompensatory fertility in bulls (semen)	795	Holstein	(18)
14	5.93 Mb	38,416 SNP	Noncompensatory fertility in bulls (semen)	795	Holstein	(18)
15	46.78 Mb	38,416 SNP	Noncompensatory fertility in bulls (semen)	795	Holstein	(18)
17	58.73 Mb	38,416 SNP	Noncompensatory fertility in bulls (semen)	795	Holstein	(18)
19	62.00 Mb	38,416 SNP	Noncompensatory fertility in bulls (semen)	795	Holstein	(18)
22	38.91 Mb	38,416 SNP	Noncompensatory fertility in bulls (semen)	795	Holstein	(18)
22	58.96 Mb	38,416 SNP	Noncompensatory fertility in bulls (semen)	795	Holstein	(18)
X	31.67 Mb	38,416 SNP	Noncompensatory fertility in bulls (semen)	795	Holstein	(18)
X	43.13 Mb	38,416 SNP	Noncompensatory fertility in bulls (semen)	795	Holstein	(18)
X	101.55 Mb	38,416 SNP	Noncompensatory fertility in bulls (semen)	795	Holstein	(18)
1	95 cM (BMS4031−91.3 Mb)	390 MS	Scrotal circumference	1,769	Angus	(19)
4	46 cM (BMS1840−51 Mb)	390 MS	Scrotal circumference	1,769	Angus	(19)
4	96 cM (RM088−108.5 Mb)	390 MS	Scrotal circumference	1,769	Angus	(19)
5	12 cM (BMS610−13 Mb)	390 MS	Scrotal circumference	1,769	Angus	(19)
5	101 cM (BM315−104 Mb)	390 MS	Scrotal circumference	1,769	Angus	(19)
5	127 cM (BMS597)	390 MS	Scrotal circumference	1,769	Angus	(19)
6	102 cM (BM8124)	390 MS	Scrotal circumference	1,769	Angus	(19)
7	10 cM (RM012−0.5 Mb)	390 MS	Scrotal circumference	1,769	Angus	(19)
7	28 cM (RM006−16 Mb)	390 MS	Scrotal circumference	1,769	Angus	(19)
7	41 cM (BM6105−22 Mb)	390 MS	Scrotal circumference	1,769	Angus	(19)
8	12 cM (IDVGA11−10 Mb)	390 MS	Scrotal circumference	1,769	Angus	(19)
9	68 cM (BMS2377−72.7 Mb)	390 MS	Scrotal circumference	1,769	Angus	(19)
9	110 cM (BMS1967−92 Mb)	390 MS	Scrotal circumference	1,769	Angus	(19)
10	99 cM (BMS614−94 Mb)	390 MS	Scrotal circumference	1,769	Angus	(19)
10	118 cM (BL1134−102 Mb)	390 MS	Scrotal circumference	1,769	Angus	(19)
11	12 cM (INRA044−6 Mb)	390 MS	Scrotal circumference	1,769	Angus	(19)
11	29 cM (BMS2325−11.8 Mb)	390 MS	Scrotal circumference	1,769	Angus	(19)
11	93 cM (BMS989−86.2 Mb)	390 MS	Scrotal circumference	1,769	Angus	(19)
12	13 cM (BMS2252−10.4 Mb)	390 MS	Scrotal circumference	1,769	Angus	(19)
13	41 cM (BMS1352−28.1 Mb)	390 MS	Scrotal circumference	1,769	Angus	(19)
15	21 cM (ADCY2-BTA20 at 69.2 Mb)	390 MS	Scrotal circumference	1,769	Angus	(19)
15	34 cM (JAB8−29.3 Mb)	390 MS	Scrotal circumference	1,769	Angus	(19)
16	73 cM (INRA048)	390 MS	Scrotal circumference	1,769	Angus	(19)
17	94 cM (BM1233-BTA18 54.7 Mb)	390 MS	Scrotal circumference	1,769	Angus	(19)
18	77 cM (BM2078−62.0 Mb)	390 MS	Scrotal circumference	1,769	Angus	(19)
19	12 cM (BMS745−11.8 Mb)	390 MS	Scrotal circumference	1,769	Angus	(19)
19	56 cM (BMS650−36.2 Mb)	390 MS	Scrotal circumference	1,769	Angus	(19)
19	80 cM (IDVGA44−56.7 Mb)	390 MS	Scrotal circumference	1,769	Angus	(19)
19	98 cM (RM388−59.4 Mb)	390 MS	Scrotal circumference	1,769	Angus	(19)
20	1 cM (RM106−1.2 Mb)	390 MS	Scrotal circumference	1,769	Angus	(19)
21	30 cM (BM103−20.0 Mb)	390 MS	Scrotal circumference	1,769	Angus	(19)
22	27 cM (DIK2694−21.1 Mb)	390 MS	Scrotal circumference	1,769	Angus	(19)
22	65 cM (BMS875−46.1 Mb)	390 MS	Scrotal circumference	1,769	Angus	(19)
23	35 cM (BOLADRB1−26.3 Mb)	390 MS	Scrotal circumference	1,769	Angus	(19)
25	59 cM (BMS1353−32.9 Mb)	390 MS	Scrotal circumference	1,769	Angus	(19)
26	15 cM (FASMC2−11.1 Mb)	390 MS	Scrotal circumference	1,769	Angus	(19)
27	61 cM (BMS1675−46.2 Mb)	390 MS	Scrotal circumference	1,769	Angus	(19)
28	30 cM (BMS510−21.8 Mb)	390 MS	Scrotal circumference	1,769	Angus	(19)
28	49 cM (BMS1714−34.6 Mb)	390 MS	Scrotal circumference	1,769	Angus	(19)
29	13 cM (BMS764−10.0 Mb)	390 MS	Scrotal circumference	1,769	Angus	(19)
2	108–109 Mb	43,821 SNP	Serum inhibin at 4 months	786	Brahman	(20)
14	22–26 Mb	43,821 SNP	Scrotal circumference at 12 months	1,112	Brahman	(20)
28	18 Mb	43,821 SNP	Luteinizing hormone levels at 4 months	537	Brahman	(20)
X	4 Mb	43,821 SNP	Percent normal sperm at 24 months	964	Brahman	(20)
X	40–55 Mb	43,821 SNP	Percent normal sperm at 24 months	964	Brahman	(20)
X	97 Mb	43,821 SNP	Percent normal sperm at 24 months	964	Brahman	(20)
X	62–96 Mb	43,821 SNP	Scrotal circumference at 12 months	1,112	Brahman	(20)
14	22–28 Mb	43,821 SNP	Age at puberty	1,118	Brahman	(21)
X	86 Mb	43,821 SNP	Age at puberty	1,118	Brahman	(21)
2	25.6 Mb	38,650 SNP	Sire conception rate	1,755	Holstein	(22)
5	119.4 Mb	38,650 SNP	Sire conception rate	1,755	Holstein	(22)
18	54.3 Mb	38,650 SNP	Sire conception rate	1,755	Holstein	(22)
25	1.4 Mb	38,650 SNP	Sire conception rate	1,755	Holstein	(22)
25	2.8 Mb	38,650 SNP	Sire conception rate	1,755	Holstein	(22)
25	4.8 Mb	38,650 SNP	Sire conception rate	1,755	Holstein	(22)
13	8.42 Mb	46,035 SNP	Tail stump sperm defect	321	Swedish Red	(23)
25	2.98 Mb	54,001 SNP	Asthenospermia		Nordic Red	(24)

a *Chromosomal positions are represented in centiMorgans (cM). The microsatellite marker location was employed to translate cM into Mb positions, according to the BTAU4.0 assembly.*

b *Indicates the number of gene markers used in the relevant study (SNP, single nucleotide polymorphisms; MS, microsatellite).*

c *Represents how many experimental animals were used*.

Epigenetics refers to molecular processes that may regulate gene expression (active vs. inactive genes) without alterations in the DNA sequence. Epigenetic modifications, including DNA methylation, histone modifications, and nucleosome positioning, can be transmitted to the daughter cells through cell divisions. Aberrant alterations in the epigenetic profiles may give rise to abnormal gene silencing or activation (28). Transformation of male germ cells into functional spermatozoa requires a specific order involving the accumulation of specific non-coding RNA, substitution of protamines for most histones, and large-scale DNA methylation changes (29, 30). Although transcription is hardly observable in the mature sperm cells, the differentiation program in the male germline is regulated through a series of transcriptional modulations that depend directly on epigenetic reprogramming (31, 32).

## Proteomics, Transcriptomics, and Metabolomics of Sperm Cells

### Sperm Proteins and Bull Fertility

Sperm contains diverse proteins present in the sperm membrane, flagellum, cytoplasm, acrosome, and nucleus that play key roles in sperm physiology (33). Of these proteins, some are energy-related enzymes involved in sperm motility, both signaling and structural. For example, the outer dense fiber protein (ODF) has been implicated in the protection of the sperm tail against shear forces and motility in the mouse (34). Zhao et al. (35) stated that ODF2 might bind to and maintain acetylated levels of α-tubulin in HEK293T cell lines exposed to cold environment. In humans, energy-related proteins isocitrate dehydrogenase subunit alpha and phosphoglycerate mutase 2 are down- or upregulated in asthenozoospermia, respectively (36). Sperm postacrosomal sheath WW domain-binding protein (PAWP) and PLC zeta are involved in oocyte activation and embryogenesis in mice and humans (37, 38). However, Satouh et al. (39), using real-time PCR, immunoblotting, and electron microscopy, asserted that PAWP does not play an essential role in the formation of mouse sperm head or spermatogenesis in PAWP null mice. Compared with other studies, the differences in the findings of Satouh et al. (39) may arise from methodological approaches used and, perhaps, the functional interaction of PAWP with other proteins.

Using Western blotting and real-time PCR, Velho et al. (40) postulated that expression of integrin subunit beta 5 (ITGβ5) in germ cells and resultant embryos is important for fertilization and embryonic development in bovine. The fertility prediction for each bull was obtained using the Probit.F90 software (41) and expressed as the percent deviation of its conception rate from the average conception rate of all bulls. Moreover, IZUMO and fertilin subunit beta (ADAM 2) is considered to play a crucial role in the interactions between the sperm and zona pellucida, and in acrosome reactions. IZUMO1 binds to Juno, a receptor present on the egg, and facilitates gamete recognition during fertilization (42). Using 2D-PAGE, Park et al. (42) showed that ATP synthase H+ transporting mitochondrial F1 complex beta subunit (ATP5B), alpha-2-HS-glycoprotein 2 (AHSG), enolase 1 (ENO1), apoptosis-stimulating of p53 protein (ASPP2), and phospholipid hydroperoxide glutathione peroxide (GPx4) were more abundant in sperm from high-fertility bulls, whereas ubiquinol–cytochrome c reductase complex core protein 2 (UQCRC2), ropporin-1, and voltage-dependent anion channel 2 (VDAC2) were in greater amounts in sperm from low-fertility bulls (Table 2).

**Table 2:** Fertility-associated proteins of sperm from low- and high-fertility bulls.

**Protein name**	**Abbreviation**	**High fertility**	**Low fertility**	**Function**	**Methods**	**Breed**	**References**
ATP synthase H+ transporting mitochondrial F1 complex beta subunit	ATP5B	Upregulated		Energy metabolism	2D-PAGE	Hanwoo	(42)
Alpha-2-HS-glycoprotein 2	AHSG	Upregulated		Immune system	2D-PAGE	Hanwoo	(42)
Enolase 1	ENO1	Upregulated		Energy metabolism	2D-PAGE	Hanwoo	(42)
Apoptosis-stimulating of p53 protein	ASPP2	Upregulated		Oxidative stress	2D-PAGE	Hanwoo	(42)
Phospholipid hydro peroxide glutathione peroxide	GPx4	Upregulated		Oxidative stress	2D-PAGE	Hanwoo	(42)
Ubiquinol-cytochrome c reductase complex core protein 2	UQCRC2		Upregulated	Oxidative stress	2D-PAGE	Hanwoo	(42)
Ropporin-1			Upregulated	Cell signaling	2D-PAGE	Hanwoo	(42)
Voltage-dependent anion channel 2	VDAC2		Upregulated	Ion transport	2D-PAGE	Hanwoo	(42)
Malate dehydrogenase 2	MD2	Upregulated		Energy metabolism	2D-DIGE and MALDI-TOF-MS	Holstein x Tharparkar crossbred	(5)
Enolase 1	ENO1	Upregulated		Energy metabolism	2D-DIGE and MALDI-TOF-MS	Holstein x Tharparkar crossbred	(5)
Calpain-7-like protein	CAPN7	Upregulated		Acrosome reaction and capacitation	2D-DIGE and MALDI-TOF-MS	Holstein x Tharparkar crossbred	(5)
N-Acetyllactosaminide beta-1,6 N acetylglucosaminyl transferase isoform C	GCNT2	Upregulated		Development and maturation of erythroid cells	2D-DIGE and MALDI-TOF-MS	Holstein x Tharparkar crossbred	(5)
RIB43A domain with coiled-coils 1	RIBC1	Upregulated		Sperm motility and the structural integrity of sperm tail	2D-DIGE and MALDI-TOF-MS	Holstein x Tharparkar crossbred	(5)
Condensen-2 complex subunit D3	NCAPD3	Upregulated			2D-DIGE and MALDI-TOF-MS	Holstein x Tharparkar crossbred	(5)
2,4-Dienoyl CoA reductase-1	DECR1	Up regulated		Energy metabolism	2D-DIGE and MALDI-TOF-MS	Holstein x Tharparkar crossbred	(5)
Beta galactosidase-1-like protein-2	LacA like protein-2	Upregulated		Maturation of spermatozoa	2D-DIGE and MALDI-TOF-MS	Holstein x Tharparkar crossbred	(5)
GDP dissociation inhibitor 2	GDI2	Upregulated		Preventing membrane integrity	2D-DIGE and MALDI-TOF-MS	Holstein x Tharparkar crossbred	(5)
Chain D, F-1 ATPase	ATP5D	Upregulated		Energy metabolism	2D-DIGE and MALDI-TOF-MS	Holstein x Tharparkar crossbred	(5)
Ubiquitin carboxyl terminal hydrolase-12	USP12	Upregulated		Cell signaling	2D-DIGE and MALDI-TOF-MS	Holstein x Tharparkar crossbred	(5)
Thimet oligopeptidase-1	TOP	Upregulated		Catalyze the hydrolysis of gonadotropin-releasing hormone	2D-DIGE and MALDI-TOF-MS	Holstein x Tharparkar crossbred	(5)
Binder of sperm-1	BSP1		Upregulated	Prevent premature acrosome reaction and capacitation	2D-DIGE and MALDI-TOF-MS	Holstein x Tharparkar crossbred	(5)
Transmembrane protein-43	TMEM43		Upregulated	Maintain nuclear envelope structure	2D-DIGE and MALDI-TOF-MS	Holstein x Tharparkar crossbred	(5)
Dystonin-like isoform-1	DST like isoform 1		Upregulated	An integrator of intermediate filaments, actin, and microtubule cytoskeleton networks	2D-DIGE and MALDI-TOF-MS	Holstein x Tharparkar crossbred	(5)
Albumin	ALB	Upregulated		Ease cholesterol outflow from sperm membranes and preserves sperm against lipid peroxidation	2D-DIGE	Holstein	(44)
The tissue inhibitors of metalloproteinase	TIMP	Upregulated		Inhibit MMPs by binding to their catalytic Zn cofactor	2D-DIGE	Holstein	(44)
Spermadhesin-1	SPADH1	Upregulated		Participate in sperm–egg binding	2D-DIGE	Holstein	(44)
Binder of sperm proteins 1, 3, and 5	BSP1, 3, 5	Upregulated		Prevent premature acrosome reaction and capacitation	2D-DIGE	Holstein	(44)
Phosphatidylethanolamine-binding protein 1	PEBP1	Upregulated		Promote inhibition of early sperm capacitation	2D-DIGE	Holstein	(44)
Adenylate kinase isoenzyme 1	AK1	Upregulated		Energy metabolism	2D-DIGE	Holstein	(44)
Heat shock protein 90	HSP90	Upregulated		Stabilizes proteins against heat stress	2D-DIGE	Holstein	(44)
B-cell lymphoma-62	BCL62	Upregulated		Antiapoptotic	2D-DIGE	Holstein	(44)
NADH dehydrogenase	NADHD	Upregulated		Energy metabolism	2D-DIGE	Holstein	(44)
Interferon regulatory factor 4	IFNRF4	Upregulated		Immune system	2D-DIGE	Holstein	(44)
Class III β-tubulin	TUBB3	Upregulated		Sperm motility	2D-DIGE	Holstein	(44)
Proteasome subunit alpha type-6	PSMA6		Upregulated	Associated with sperm DNA fragmentation	2D-DIGE	Holstein	(44)
Phosphatidylethanolamine-binding protein 1	PEBP1		Upregulated	Inhibition of sperm capacitation	2D-DIGE	Holstein	(44)
T-complex protein 1 subunits 3 and 8	CCT3, CCT8		Upregulated	Reflecting incomplete developmental processes	2D-DIGE	Holstein	(44)
Clusterin	CLU		Upregulated	Oxidative stress	2D-DIGE	Holstein	(44)
The tissue inhibitors of metalloproteinase-2	TIMP-2	Upregulated		Inhibit MMPs by binding to their catalytic Zn cofactor	Mass spectrometry coupled with Nano HPLC	Holstein	(4)
C-type natriuretic peptide	NPPC	Upregulated		Stimulating intracellular cGMP and sperm motility	Mass spectrometry coupled with Nano HPLC	Holstein	(4)
Sulfhydryl oxidase	QSOX1	Upregulated		Oxidative stress	Mass spectrometry coupled with Nano HPLC	Holstein	(4)
Binder of sperm-5	BSP5	Upregulated		Participate in sperm–egg binding	Mass spectrometry coupled with Nano HPLC	Holstein	(4)
Galectin-3-binding protein	LGALS3BP		Upregulated	Inhibiting cell signaling	Mass spectrometry coupled with Nano HPLC	Holstein	(4)
Tissue factor pathway inhibitor 2	TFPI2		Upregulated		Mass spectrometry coupled with Nano HPLC	Holstein	(4)
Clusterin	CLU		Upregulated	Oxidative stress	Mass spectrometry coupled with Nano HPLC	Holstein	(4)

Currently, proteomic approaches are widely used to explore male reproductive physiology (43). Aslam et al. (5) analyzed the bull sperm proteome using 2D-DIGE and MALDI-TOF-MS techniques, and validated these proteomic studies using Western blotting. The authors reported that malate dehydrogenase 2 (MD2), enolase 1 (ENO1), calpain-7 like protein (CAPN7), N-acetyllactosaminide beta-1,6-N-acetylglucosaminyl transferase isoform C (GCNT2), RIB43A domain with coiled-coils 1 (RIBC1), condensen-2 complex subunit D3 (NCAPD3), 2,4-dienoyl CoA reductase-1 (DECR1), beta galactosidase-1-like protein-2 like (LacA-like protein-2 like), GDP dissociation inhibitor 2 (GDI2), chain D, F-1 ATPase (ATP5D), ubiquitin carboxyl terminal hydrolase-12 (USP12), and thimet oligopeptidase-1 (TOP) are over expressed in sperm from high-fertility bulls, whereas binder of sperm-1 (BSP1), transmembrane protein-43 (TMEM43), and dystonin-like isoform-1(DST like isoform 1) are more abundant in sperm from low-fertility bulls (Table 2).

The MDH2 catalyzes the reversible oxidation of malate to oxaloacetate using NAD+/NADH as a cofactor in the citric acid cycle (45). Aslam et al. (5) suggested that the reduction of MDH2 has a negative impact on energy metabolism of spermatozoa, disrupting sperm motility, capacitation, and ultimately fertilizing ability. ENO1, a multifunctional enzyme, is found mainly in the motile sperm tail. In addition to regulating the constant provision of energy for motility, it assists in the protection of the sperm from oxidative stress (42). The RibC is a ribbon protein that is vital for sperm motility and structural integrity of sperm tails, suggesting that low expression of RibC in bull sperm reduces fertility by disrupting sperm motility (5). Calpains in mammalian sperm are involved during the acrosome reaction and capacitation (46). The Rab are small GTP-binding proteins that are critical in vesicular trafficking of molecules. The GDI keeps the function of Rab proteins under control by freeing it from membranes and preventing the GDP dissociation (47). The USP12 plays a crucial role in maintaining the androgen receptors steady and improving their cellular functions (48). The TOP are highly expressed enzymes in testes and exert their functions by catalyzing the hydrolysis of gonadotropin-releasing hormone (49). The LacA-like protein-2, which is produced and secreted from the epididymis, binds to sperm membranes during the maturation process in rats (50). Aslam et al. (5) suggested that low levels of expression of this enzymatic protein are considered to have a significant role in sperm physiology and led to a reduction in functional competence of the sperm in low-fertility bulls.

Using 2D-DIGE analysis of bull sperm, it was shown that ALB, TIMP, spermadhesin-1, and binder of sperm proteins (BSP)-1, 3, and 5, PEBP1, and AKI in sperm and seminal plasma were more abundant in sperm from high fertility bulls, while PSMA6, ELSPbP1, CCT5, CCT8, and CLU were in greater amounts in seminal plasma and in sperm from low-fertility bulls. The expression levels of ZFP34, HSP90, BCL62, IFNRF4, NADHD, histone H1, and TUBB3 were higher in high-fertility bull sperm (44) (Table 2). Matrix metalloproteinases (MMPs) belonging to a group of proteolytic zinc-dependent enzymes are crucial components of semen (51). The MMPs and other proteases participate in semen liquefaction in the female genital tracts, and they are needed for sperm viability during capacitation in humans (52).

Spermadhesin family members interact with carbohydrates, phospholipids, and zona pellucida glycoproteins and participate in sperm–egg binding (53–55). Spermadhesin-1 is a nonglycosylated protein produced by the epithelium of the epididymis, ampulla, and seminal vesicle, and is secreted into the seminal fluid (56, 57). Furthermore, it has been suggested that recombinant spermadhesin-1 influences sperm mitochondrial activity through its binding ability to the sperm midpiece (58).

Albumin has been reported to facilitate cholesterol outflow from sperm membranes and mediates sperm capacitation in the female reproductive tract (59, 60). Moreover, albumin preserves sperm against lipid peroxidation by binding to free radicals (44). Adenylate kinase isoenzyme 1 (AK1), a ubiquitous enzyme related to cellular energy homeostasis, is expressed in murine and bovine sperm flagella, suggesting its participation in sperm motility (61–63). Furthermore, AK1 has been reported to be active when spermatozoa are highly motile (62). Phosphatidylethanolamine-binding protein 1 (PEBP1) is an evolutionarily conserved protein in mammals and reported to be present in the acrosome, the postacrosomal region, and the tail of both human and mouse sperm. The PEBP1 seems to promote inhibition of sperm capacitation because it serves either as a decapacitation factor released throughout capacitation or as a membrane-bound, glycophosphatidylinositol (GPI)-anchored receptor for a decapacitation factor (64, 65). Binder of sperm proteins (BSP) are synthesized in the male accessory sex glands and bind to sperm via choline phospholipids upon ejaculation, which prevent premature initiation of the capacitation and acrosome reaction (66). Among the BSP proteins, BSP1, BSP3, and BSP5 are predominant proteins secreted into bovine seminal plasma, all of which contain two tandem repeated fibronectin type 2 (Fn2) domains (67, 68).

Cholesterol and phospholipids contribute to the regulation of sperm membrane bilayer stability and fluidity. The BSP proteins promote efflux of phospholipids and cholesterol from sperm membranes, thereby, disrupting sperm membrane architecture, resulting in capacitation (66). Moreover, BSP proteins promote the binding of sperm to the epithelium of the oviduct, contributing to maintain sperm viability and motility in the oviduct (69). Studies on BSPs have reported different results. Some studies (5, 68, 69) showed that BSP protein expression in semen was negatively correlated with bull fertility, unlike the findings of Kasimanickam et al. (44) who reported a positive correlation between BSP expression and bull fertility. These differences were attributed to degenerated and fragmented sperm membrane wastes in semen. A single-cell analysis approach may be required to obtain a reliable result. Furthermore, due to structural similarities to BSP, epididymal sperm-binding protein E12 (ELSPbP1) can induce lipid efflux and perturb the membrane stability (70).

Proteasome subunit alpha type-6 (PSMA6) belongs to proteasome multicatalytic protease degrading polyubiquitinated proteins into small peptides and amino acids (71). Proteasomes are localized in the acrosomal region, connecting head and tail (33, 72). The presence and expression levels of PSMA6 are associated with sperm DNA fragmentation in bulls (68). T-complex protein 1 subunit 3 (CCT3) and 8 (CCT8) are parts of the class II chaperonins (73). Cytoplasmic CCT expression has been shown to localize in the centrosomes and microtubules of the manchette during spermatogenesis and assumed to be discarded during spermiation. Hence, it is considered that the abundance of CCT subunits in sperm from low-fertility bulls reflects uncompleted developmental processes throughout spermatogenesis (74). Clusterin (CLU), a 75- to 80-kDa disulfide-linked heterodimeric protein, is produced in the testis, epididymis, and seminal vesicles and has been speculated to be an alternative oxidative stress marker for seminal plasma in humans (75). The CLU is localized mainly on the abnormal sperm surface (76). Furthermore, increased levels of CLU expression in semen are positively correlated with sperm DNA defects (75).

Using mass spectrometry coupled with nano HPLC, a total of 1,159 proteins were detected in the bull seminal plasma, of which 29 were abundant in low-fertility bulls, whereas 50 were abundant in high-fertility bulls (77). While TIMP-2, C-type natriuretic peptide, sulfhydryl oxidase, and BSP5 revealed a relationship with high-fertility bulls, galectin-3-binding protein, tissue factor pathway inhibitor 2, clusterin, and 5′-nucleotidase were associated with low-fertile bulls based on multivariate analysis. Furthermore, high levels of transmembrane protein 2, prosaposin, and NAD (P) (+)-arginine ADP ribosyltransferase proteins had the highest positive correlations with fertility ranking, whereas quantities of nucleotide exchange factor SIL1, galectin-3-binding protein, and vitamin D-binding protein exhibited the highest negative correlations with fertility ranking (77) (Table 2). The C-type natriuretic peptide (NPPC) is a member of natriuretic peptides that exerts its physiological functions through binding to two distinct membrane-bound guanylyl cyclases and activating cyclic guanosine monophosphate signaling pathways (78, 79). In addition to being synthesized by cardiomyocytes and known to modulate vascular permeability and dilation/constriction, NPPC is also produced locally by Sertoli cells in the testis and serves in an autocrine manner (80). Also, NPPC is more abundantly expressed in male reproductive tissues than in other tissues (81, 82). In rats, NPPC was intensely expressed in Leydig cells and epididymal epithelium, and its expression dramatically increased after puberty (83). Furthermore, NPPC receptor (NPR-B) has been shown to localize in the acrosome and tail of human sperm, suggesting that NPPC binds to NPR-B, thus, stimulating intracellular cGMP and sperm motility (84). In the male reproductive tract, QSOX1 maintains the structure and function of sperm through the oxidization of sulfhydryl groups that might damage the cell (85). Sulfhydryl oxidase (QSOX1) is involved in the reduction of an oxygen molecule to hydrogen peroxide; thus, it creates disulfide bonds in peptides and proteins (86). It has been proposed that QSOX is essential for sperm physiology, and its dysregulation is attributed to defects that may occur during spermatogenesis in hamsters (87) and rats (88).

Galectins (Gals) belong to members of ß-galactoside-binding lectins, which can be localized in extracellular spaces and in cellular components such as cell membrane, cytoplasm, and nucleus (89). They are implicated in cell-to-cell interactions, cell–extracellular matrix interactions, receptor crosslinking or lattice formation, intracellular signaling, and posttranscriptional splicing (90). Gal-3 has antiapoptotic effects, unlike most members of the galectin family (91). Gal-3 expression has been observed in the epithelium of corpus and cauda epididymis but not in initial segment and caput epididymis, suggesting that Gal-3 participates in maturation and storage of rat sperm (92). Previously, Gal-3-binding protein has been observed in bovine epidydimal fluid (57) and shown to participate in sperm motility, semen liquefaction, and angiogenesis in the female reproductive tract (93).

Gomes et al. (94) examined the proteome and posttranslational modifications in bovine seminal plasma with the aid of a top–down mass spectrometry (TDMS) strategy to uncover more comprehensive information. They separated plasma proteins using sheathless capillary zone electrophoresis (CZE)-MS and reversed-phase liquid chromatography (LC)-MS. Then, the proteins were fragmented using electron-transfer/higher-energy collisional dissociation and 213-nm ultraviolet photodissociation. The use of the sheathless CZE-MS method helped identify 417 proteoforms, including 170 unique species, whereas 3,090 proteoforms, including 1,707 unique species were detected by using LC-MS. The researchers identified 1,433 proteoforms (EThcD) and 2,151 proteoforms (213 nm UVPD) with 612 species for EThcD and 1,021 for 213-nm UVPD (94).

### Sperm Transcriptome and Bull Fertility

Sperm delivers, not just the paternal DNA, but other factors, such as cell signaling molecules, RNA, and transcription factors, into the oocyte at the time of fertilization (95). New cutting-edge technologies, such as RNA sequencing (RNA-seq) and microarray analysis have enabled characterization of various types of sperm RNAs, including transfer RNA (tRNA), ribosomal RNA (rRNA), messenger RNA (mRNA), small nucleolar RNA (snoRNA), small nuclear RNA (snRNA), small non-coding RNA (sncRNA), long non-coding RNA (lncRNA), and mitochondrial RNA (mt-RNA), which are present in bovine spermatozoa (96). The miRNAs, piRNAs, and tRNAs are grouped as “small non-coding” RNAs (sRNAs) (97).

There are several transcriptomic studies on bull sperm using different techniques. Wang et al. (98) used strand-specific RNA sequencing to profile the semen transcriptome (lncRNA and mRNA) and to ascertain the functions of lncRNA and mRNA in bull sperm motility. They detected 20,875 transcripts of protein-encoding genes in semen and found 19 different mRNAs between high- and low-motility sperm. They also detected five differentially expressed genes, such as *Efna1, Rbmx, Mlph, Rpl30*, and *Aqp2*, which participate in “extracellular exosome” GO term. Among them, the ephrin A1 (Efna1) protein that is localized on cell surfaces participates in membrane integrity and sperm morphology, and it has been reported that *Efna1* is highly present in both seminal plasma and sperm (44, 99) and possibly influences sperm motility (98). Heterogeneous nuclear ribonucleoprotein G (*Rbmx*) has been proposed to be a possible splicing factor that modulates spermatogenesis (100). Based on immunohistochemical methods, aquaporin 2 (*Aqp2*) is expressed in male germ cells, seminiferous epithelium, Leydig cells, and in the male reproductive tract (101), suggesting that *Aqp2* directly or indirectly participates in male fertility.

Moreover, Wang et al. (98) also identified 11,561 lncRNA in bull sperm, of which 2,517 were distinctly expressed between the low- and high-motility sperm. They also determined that TCONS_00041733 lncRNA targets the node gene *ephrin A1* and participates in the physiology of the male reproductive system. Card et al. (102) detected 6,166 transcripts in bull sperm, most of which were full-length transcripts that *Plcz1* and *Crisp2* transcripts are associated with bull fertility. Furthermore, a comprehensive microarray analysis revealed 415 transcripts to be differentially expressed in sperm from high- and low-fertility bulls (103). Légaré et al. (104) showed that 10 mRNA transcripts (*Smcp, Akap4, Tcp11, Spata3, Ctcfl, Odf1, Adam28, Spata18, Fam161a*, and *Sord*) in bovine sperm were associated with reproductive system functions. They also found that five mRNA transcripts (*Cyst11, Dead, Mx1, Defb124*, and *Defb119*) are related to the immune defense response.

Sperm miRNA content is dynamic, and the factors affecting spermatogenesis and epididymal maturation influence sperm miRNA composition (105, 106). Microarray and RNA-Seq-based gene expression profiling studies showed that *miR-10a, miR-10b, miR-34c, miR-100, miR-103, miR-196b, miR-365-2*, and *miR- 2478* consistently exist in bovine spermatozoa (96, 105, 107). Interestingly, RT-qPCR studies determined that *miR-19b-3p, miR-34c-3p, miR-148b-3p, miR-320a*, and *miRNAs miR-1249* were detectable at low levels, whereas *miR-27a-5p* and *502-5p* were not detectable in sperm from most high-fertility bulls (108). These miRNA transcripts, such as *miR-34, miR-34b/c*, and *miR-449*, modulate spermatogenesis and possibly embryogenesis (109, 110). Liu et al. (111) have suggested that *miR-34c-5p* is involved in mRNA degradation and translational repression.

Two different RNA-seq platforms were Illumina and Ion Proton, and they provided evidence that the most abundant miRNA in the bovine sperm is *miR-196b* and is more abundantly expressed in the zygote than the oocyte. *miR-196b* targets transcripts of *Hoxa7, Hoxa9*, and *Hoxc8* genes. In addition, protein products of these genes play crucial roles in the meiotic phases of spermatogenesis and are present at high levels in spermatocytes (96, 111, 112). Menezes et al. (113) examined the dynamics of *miR-15a, miR-29b*, and *miR-34a* in low- and high-fertility bull sperm using RT-qPCR (113). They reported that *miR-15a* and *miR-29* were more abundantly present in sperm from low-fertility bulls than those of high fertility bulls. However, *miR-34a* expression levels did not differ in sperm from the two groups. In addition, results of several studies suggest that lncRNAs may be involved in the regulation of testis development and spermatogenesis. For example, Zhang et al. (114) showed that *Dmrt1* was involved in the transition of germ cells from mitosis to meiosis using transfection, Western blotting, and Northern and Southern blotting hybridizations. Based on proteomic, immunostaining, and microarray approaches, *HongrES2* has been reported to modulate sperm maturation, and *Mrhl* lncRNA influences spermatogenesis (115, 116). The *Tsx*, which is specifically expressed in pachytene spermatocytes, has a crucial role in the progression of spermatocyte meiosis (117).

### Sperm Metabolome and Bull Fertility

Metabolites are also associated with physiological events via a cascade of complex biochemical networks (118, 119) and may provide insights of an individual's phenom (119). Metabolomic methods are used to detect low molecular weight compounds that may offer deep insights into the regulatory pathways within spermatozoa as well (120, 121). In this regard, the mounting evidence shows that mature sperm metabolize a wide range of exogenous substrates that modulate the signaling pathways implicated in key aspects of sperm physiology, including the acrosome reaction, capacitation, hyperactivation, motility, and fusion of spermatozoon and egg (122). The latest improvements in methods of metabolite profiling of infertile individuals offer better insights into the development of useful fertility markers (123). Several metabolite biomarkers have been discovered by untargeted metabolic profiling of sperm samples from healthy individuals and infertile patients using different analytical techniques such as nuclear magnetic resonance (NMR) (124) and mass spectrometry (MS) (125).

There were 22 distinct metabolites detectable in bull sperm employing gas chromatography-mass spectrometry (GC-MS) analysis (126) where major metabolites were fatty acids/conjugates and organic acids/derivatives. The researchers also showed that the levels of five sperm metabolites that differed between high- *vs*. low-fertility groups were benzoic acid, gamma-aminobutyric acid (GABA), palmitic acid, carbamate, and lactic acid. In addition, four metabolic pathways were found to be associated with differential metabolites, namely, glycolysis or gluconeogenesis, aspartate and glutamate metabolism, pyruvate metabolism, alanine, and β-alanine metabolism. GABA plays an essential role in sperm physiology by inducing the acrosome reaction and sperm hyperactivation. Furthermore, benzoic acid participates in GABA regulation and is highly expressed in high-fertility bull sperm. Because of its participation in lipid metabolism to generate energy, palmitic acid production may be more abundant in high-fertility bull sperm. Higher levels of lactic acid in high-fertility bull sperm might be because anaerobic glycolysis is more efficiently utilized in high-fertility sperm compared with those in low-fertility sperm. Functions of carbamate are considered as potential regulators of intracellular pH in sperm (127).

Velho et al. (128) studied seminal plasma metabolomes of Holstein bulls using GC-MS. They reported that the most abundant metabolites were fructose followed by urea, citric acid, phosphoric acid, and lactic acid. Erythronic acid, 4-ketoglucose, 2-oxoglutaric acid, androstenedione, and D-xylofuranose represented the least predominant metabolites in bull seminal fluid. They demonstrated that levels of 2-oxoglutaric acid were low, whereas the levels of fructose were greater in high-fertility bulls compared with low-fertility bulls. Sperm metabolism can oxidize fructose and convert it to lactic acid (129), supporting both fructose and lactic acid as necessary for fertile sperm as energy sources. Therefore, in many species, fructose is the main monosaccharide abundantly present in semen (124, 130–132).

Citric acid influences the acrosome reaction, sperm transport, and fertilization by being an energy source and regulating semen pH as a chelator for calcium, magnesium, and zinc (133, 134), suggesting that citric acid is a candidate fertility marker in seminal plasma. However, roles of urea and phosphoric acid in seminal plasma on bull fertility remain mostly unclear. Velho et al. (128) speculated that phosphoric acid in seminal plasma may result from catalysis of inorganic phosphate. Hydrolysis of inorganic pyrophosphate to two phosphate ions yields energy (135) that may be utilized for sperm motility or fertilization. Urea in seminal plasma is considered as a metabolite resulting from protein degradation (136). High concentrations of urea in seminal plasma suggest that fertile sperm contain enough protein sources, and some of these proteins are metabolized for biological processes associated with fertility. Using MS, Soggiu et al. (137) demonstrated that isocitrate dehydrogenase, triose phosphate isomerase, and alpha enolase were fertility-associated molecules in bull sperm. Recently, amino acid contents in seminal plasma were shown to be associated with bull sperm freezability (138). Using GC-MS, the researchers also showed that the most abundant amino acid in bull seminal fluid was glutamic acid. Furthermore, phenylalanine concentration in seminal plasma was significantly associated with post-thaw viability.

## Sperm Epigenome and Bull Fertility

### Sperm DNA Methylation and Bull Fertility

DNA methylation has been the most studied epigenetic mechanism in sperm and is presumed to fulfill a major role in the non-genetic information transfer across generations. Sperm DNA methylation participates in many physiological processes, such as silencing of transposable elements (139), paternal genomic imprinting (30), DNA compaction (140), and chromosome inactivation in females (141). In combination with histone modifications, DNA methylation has a fundamental role in modulating gene expression in germ cells by inhibiting the binding of transcription factors to enhancers or by recruiting the binding of proteins that facilitate the deacetylation or methylation of histones, thereby stabilizing the nucleosomes (142). Advances in technologies offered quantitative and base-level ultra-resolution methylome maps. DNA methylation involves the addition of a methyl group to the carbon-5 position of cytosine in the context of cytosine followed by guanine (CpG dinucleotides), referred to as 5-methylcytosine (5mC), although to a lesser extent, DNA methylation also occurs at cytosine bases in a non-CpG context (143, 144).

Gametic DNA methylation is archived in a progressive manner via the activity of the *de novo* methyltransferases DNMT3A, DNMT3B, and their cofactor DNMT3L. Significant levels of DNA methylation are present at birth and must be sustained by DNMT1 during adulthood across different phases of spermatogenesis (145, 146). However, DNA methylation can be reversible, mediated by the ten-eleven translocation (TET) family of DNA dioxygenases that progressively oxidize 5mC to 5-hydroxymethylcytosine (5hmC), 5-formylcytosine (5fC), and 5-carboxylcytosine (5caC) (147–151). The CpG islands experience differential methylation during gametogenesis and early embryonic development (152). Exposure to harmful environmental conditions may alter DNA methylation patterns in male germ cells and inhibit differentiation into functional mature spermatozoa, thereby causing infertility (153, 154).

Employing whole-genome bisulfite sequencing (WGBS) data (486 × coverage) from neighboring CpG sites among 28 distinct bull sperm samples, Liu et al. (155) identified 31,272 methylation haplotype blocks (MHB) based on the correlation analysis of methylation levels. Of these MHBs, they defined highly variably methylated, variably methylated, and conserved methylated regions. By integrating evidence from traditional and molecular quantitative trait loci, they revealed that highly variably methylated regions may play roles in transcriptional regulation and function in variations in complex traits. Furthermore, they detected 46 variably methylated regions significantly related to reproduction traits, nine of which were modulated by cis-SNP. These variably methylated regions were colocalized with fertility-associated genes, such as *Crisp2, Hgf* , and *Zfp36l1*. Sperm protein CRISP2 has important roles in spermatogenesis, modulation of flagellar motility, acrosome reaction, and gamete fusion. Naz et al. (156) showed that HGF was distinctly expressed in the vas deferens and epididymis in mice. Moreover, Herness and Naz (157) implicated that HGF is involved in the process of acquisition of the potential for sperm motility as sperm mature during epididymal transit, as when immotile mouse sperm from the caput epididymis were incubated with HGF, motility of these spermatozoa was increased by 5–15%. Therefore, it is plausible that there is a relationship between expression patterns of these genes and fertility.

Using GWAS, Fang et al. (158) compared sperm DNA methylomes between cattle and humans, finding that genes with conserved hypermethylated promoters (e.g., Cd80 and Tcap) have been shown to be involved in immune responses, whereas genes with conserved non-methylated promoters (e.g., Anks1a and Wnt7a) participated in embryonic and fetal development. They also found that genes with cattle-specific hypomethylated promoters (e.g., *Dgat2* and *Ldhb*) predominantly engaged in lipid storage and metabolism (158). Using WGBS, Zhou et al. (159) compared methylomes of sperm DNA with those of three somatic tissues in bulls. They detected large differences in the methylation patterns of global CpGs, hypomethylated regions (HMR), partially methylated domains (PMD), common repeats, and pericentromeric satellites between sperm and somatic tissues. Moreover, they observed high methylation in the active gene bodies and low methylation in the promoter regions. Interestingly, meiosis-related genes including *Kif2b* and *Repin1* have been shown to be hypermethylated in somatic cells but hypomethylated in sperm. It has been reported that a broad range of kinesins have important functions in spermatogenesis. Kinesin-13 proteins, mitotic centromere-associated kinesin (MCAK), KIF2A, and KIF2B are involved in spindle bipolarity through induction of depolymerization of microtubules to modulate mitotic dynamics during spermatogenesis (160). In addition, REPIN1 could be regarded as the possible key transcription factors in spermatids (112). Therefore, previous studies support the positive correlation between hypomethylated *Kif2b and Repin1* genes with fertility. Therefore, there is a need for further studies on the functional associations between sperm DNA methylation and bull fertility and early development.

### Sperm Chromatin Dynamics and Bull Fertility

**Figure 1:**
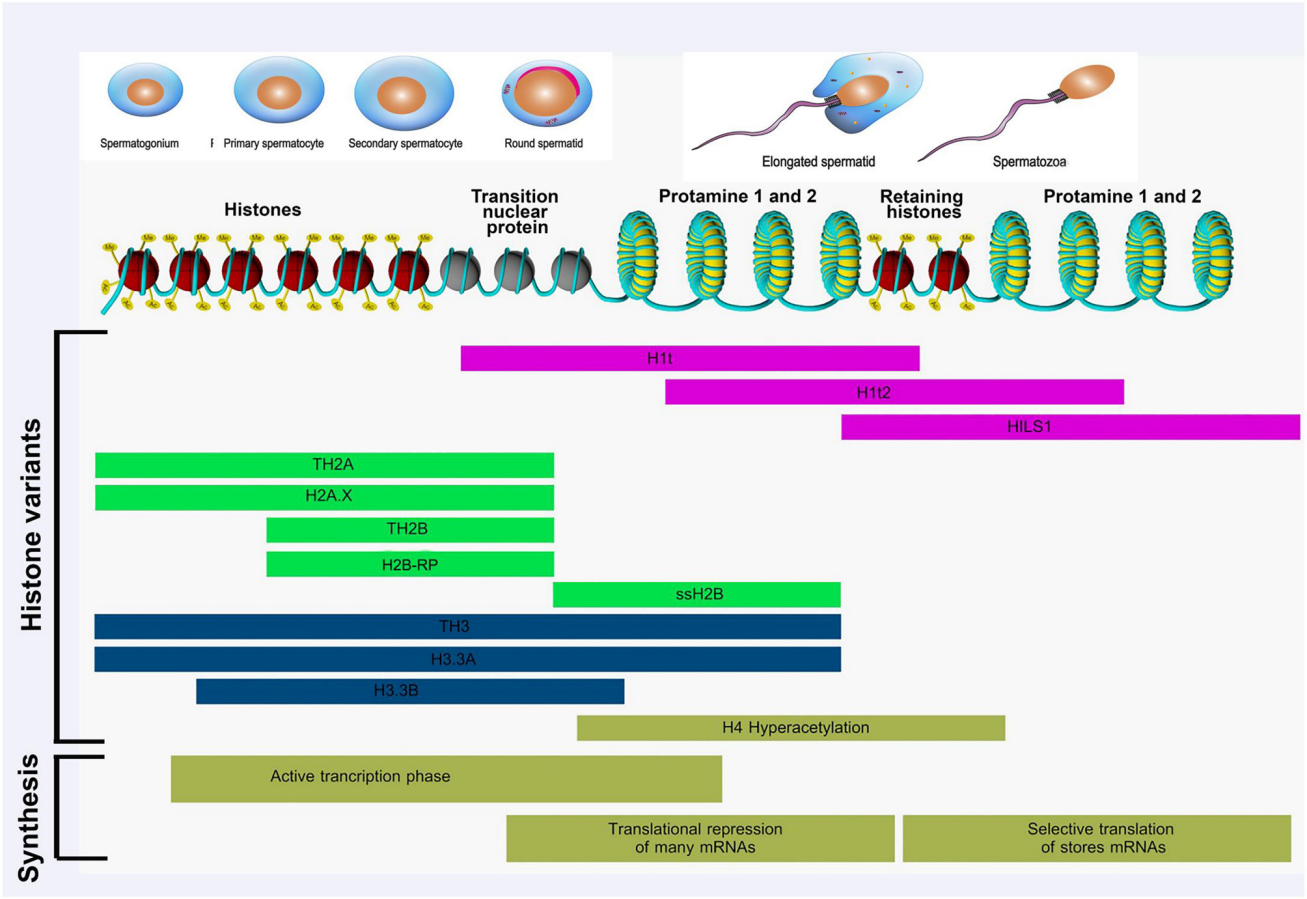
Histone modifications occurring during spermatogenesis from spermatogonia to spermatozoa. Different histone variants are transcribed and translated in this process. Active transcription is observed at the beginning of spermatogenesis. Subsequently, translation of many mRNA is repressed, stored mRNAs are repressed, and stored mRNAs are selectively translated to relevant proteins. Adapted from Kimmins and Sassone-Corsi (77) and Rathke et al. (161).

During spermiogenesis, chromatin structure and cellular morphology of round spermatids undergo dramatic reconfigurations, giving rise to an extremely condensed chromatin state and transcriptional quiescence in spermatozoa. During this period, histone hyperacetylation occurs increasingly in round and elongating spermatids, resulting in chromatin destabilization and loosening of chromatin structure to facilitate histone eviction (161, 162). In the early post-meiotic phase, most of the nucleosomal canonical histones are gradually replaced with testis-specific histone variants (noncanonical) (77). The linker histone H1 and H2A, H2B, and H3 have testis-specific histone variants. However, no histone H4 variant is known in mammals (161) (Figure 1).

Oliveira et al. (163) examined the differences in expression of two core histones (H2B and H4) and a histone variant (H3.3) in bull sperm using immunocytochemistry staining and Western blotting. However, they did not observe any differences in the levels of H2B, H3.3 or H4 in sperm from high-fertile *vs*. low-fertile bulls. Using immunofluorescence, Western blotting, and flow cytometry, Kutchy et al. (164) determined the associations between expression of the testis-specific histone variant 2B in sperm and bull fertility. Moreover, sperm chromatin damage and abnormal protamination were reported to be associated with reduced fertility in bulls using immunofluorescence, Western blotting, and chromatin dispersion tests (165, 166). In addition, using flow cytometry and immunocytochemistry, methylation and acetylation of sperm histone 3 lysine 27 (H3K27me3 and H3K27ac) were shown to be associated with bull fertility (167). Verma et al. (168) examined tri-methylated H3K27 (H3K27me3)- and di-methylated H3K4 (H3K4me2)-enriched genes in sperm of water buffalo bulls (*Bubalus bubalis*) with different fertility by using a custom ChIP on-chip array. For H3K27me3- and H3K4me2-enriched genes, they detected 80 and 84 genes, respectively. Among the H3K4me2-enriched genes, *Cct5, Cdc45, Dmc1, Meg3, Mlh1, Prdm14, Pax3, Sox4, Sox14*, and *Tbx15* have crucial roles in spermatogenesis and embryogenesis. While *Cct5, Cdc45, Dmc1, Mlh1, Prdm14, Pax3, Sox4, Sox14*, and *Tbx15* genes were in greater amounts in sperm from high fertility bulls, *Meg3* was enriched in sperm from subfertile bulls.

Considering that the H3K4me2 epigenetic modification activates gene transcription, the appearance of some H3K4me2-enriched genes in high-fertility bulls raises contradictions with previous studies. For example, CCT5 has been reported to be expressed in the microtubules of the manchette and centrosomes of spermatids and is discarded at later stages of development in mice (74). Also, CCT5 is highly expressed in sperm from low-fertility bulls (44). This might be due to the presence of seminal plasma in the samples analyzed because discarded CCT5 may be seen in the ejaculate. *Pax3, Sox4*, and *Sox14* are genes encoding for transcription factors that participate in supporting embryonic development (169, 170). The DMC1 and CDC45 are involved in meiotic recombination and initiation of chromosomal DNA replication, respectively (171, 172). Sancar (173) reported that MLH1 prevents exonuclease-mediated DNA degradation by repairing mismatched DNA pairs. Furthermore, Ji et al. (174) demonstrated that SNP in *Mlh1* gene gave rise to reduced fertility in humans. PRDM14 functions as a transcriptional regulator during germ cell development (175, 176). It also has a critical role in epigenetic modification by both recruiting DNA demethylases of the TET family and by repressing DNA methyltransferases in primordial germ cells and naïve pluripotent stem cells (177–179). Some studies reported that *Prdm14* knockout in mice gave rise to misregulation of H3K27me3 in primordial germ cells and embryonic stem cells, thereby being involved in histone modification (180, 181).

The product of *Meg3(Gtl2)* gene acts as a long non-coding RNA; therefore, it does not encode a protein. Researchers claimed that *Meg3* is involved in p53-mediated transactivation and its suppression of cell proliferation (182). Moreover, abnormal methylation of *Meg3* gene gave rise to deterioration of spermatogenesis (183). Nine of H3K27me3-enriched genes, including *Cdkn2c, Fancl, Foxa1, Gfra1, Lhx3, Rpl3, Six6, Sox4*, and *Sox14*, were speculated to be participating in sperm function and embryonic development. While H3K27me3-enriched *Foxa1* gene was in greater amounts in sperm from subfertility bulls, the others were enriched in sperm from high-fertility bulls (168). Hammoud et al. (183) showed that increase in H3K27me3 in sperm genome gives rise to inactivation of gene promoters in early embryo development. Interestingly, *Sox4* and *Sox14* genes have bivalent chromatin structure marks, both of which bear H3K27me3 and H3K4me2. Bernstein et al. (184) reported that bivalent chromatin structure marks were critical in embryonic development. On the other hand, *Cdkn2c* gene suppression is required for effective modulation of spermatogenesis in mice (185), which agrees with the reports by Verma et al. (168). There is a need for further research aimed at demystifying the functional underpinnings of suppression of these genes associated with H3K27me3 modification and fertility.

## Conclusions and Prospects

The major advancements in the *-omic* technologies (metabolomics, proteomics, transcriptomics, and genomics) have enabled high-throughput screening of a wide range of molecular and cellular dynamics in fertility molecules. These approaches also provide means of detecting minute amounts of changes in molecules due to their higher sensitivity. Such attributes of the advanced methods are vitally important for innovative studies to produce new knowledge with transformational and translational values. However, an ejaculate contains many spermatozoa with different phenotypes. Therefore, each spermatozoon should be examined using new high technologies of single-cell analyses such as single-cell metabolomics, proteomics, transcriptomics, and genomics. In addition, these methods should be combined with conventional techniques, such as sperm chromatin structure assay, computer-assisted sperm analyses (CASA), integrity of membranes, flow cytometry, and reactive oxidation stress levels, to determine semen quality using system biology approaches. As an economically important trait, fertility has become more important as there is an urgent need for more efficient, sustainable, and profitable production for food animals to feed the ever-increasing human population in the world. The bull is a unique model for the study of male fertility because of the availability of large amounts of sperm from bulls with reliable fertility phenotypes, and the significant similarities between the bull and other mammals in both sperm biology and genetics.

## Author Contributions

All authors assisted in the conception of the study, contributed to manuscript revision, read, and approved the submitted version.

## Conflict of Interest

The authors declare that the research was conducted in the absence of any commercial or financial relationships that could be construed as a potential conflict of interest.

## References

[1] FAO. Food and Agriculture Organization of the United Nations. OECD-FAO Agric Outlook. Rome: FAO (2012).

[2] Gifford JAH Gifford CA. Role of reproductive biotechnologies in enhancing food security and sustainability. Anim Front. (2013) 3:14–9. 10.2527/af.2013-0019

[3] Kaya A Memili E. Sperm macromolecules associated with bull fertility. Anim Reprod Sci. (2016) 169:88–94. 10.1016/j.anireprosci.2016.02.015 26925808

[4] Viana AGA Martins AMA Pontes AH Fontes W Castro MS Ricart CAO. Proteomic landscape of seminal plasma associated with dairy bull fertility. Sci Rep. (2018) 8:16323. 10.1038/s41598-018-34152-w 30397208 PMC6218504

[5] Aslam MMK Sharma VK Pandey S Kumaresan A Srinivasan A Datta TK. Identification of biomarker candidates for fertility in spermatozoa of crossbred bulls through comparative proteomics. Theriogenology. (2018) 119:43–51. 10.1016/j.theriogenology.2018.06.021 29982135

[6] Ugur MR Kutchy NA Menezes EB Ul-Husna A Haynes BP Uzun A. Retained acetylated histone four in bull sperm associated with fertility. Front Vet Sci. (2019) 6:223. 10.3389/fvets.2019.00223 31417913 PMC6685445

[7] Kastelic JP Thundathil JC. Breeding soundness evaluation and semen analysis for predicting bull fertility. Reprod Domest Anim. (2008) 43:368–73. 10.1111/j.1439-0531.2008.01186.x 18638148

[8] Selvaraju S Parthipan S Somashekar L Krishnan Binsila B Kolte AP Arangasamy A. Systems Biology in Reproductive Medicine Current status of sperm functional genomics and its diagnostic potential of fertility in bovine (Bos taurus). Reprod Med. (2018) 64:484–501. 10.1080/19396368.2018.1444816 29537884

[9] Staub C Johnson L. Review: spermatogenesis in the bull. Animal. (2018) 12:S27–35. 10.1017/S1751731118000435 29882505

[10] De Jonge CJ Barratt C eds. The Sperm Cell Production, Maturation, Fertilization, Regeneration. 2nd ed. Cambridge: Cambridge University Press (2017).

[11] Ugur MR Saber Abdelrahman A Evans HC Gilmore AA Hitit M Arifiantini RI. Advances in cryopreservation of bull sperm. Front Vet Sci. (2019) 6:268. 10.3389/fvets.2019.00268 31552277 PMC6736622

[12] Manolio TA Collins FS Cox NJ Goldstein DB Hindorff LA Hunter DJ. Finding the missing heritability of complex diseases. Nature. (2009) 461:747–53. 10.1038/nature08494 19812666 PMC2831613

[13] Kühn C Bennewitz J Reinsch N Xu N Thomsen H Looft C. Quantitative trait loci mapping of functional traits in the German Holstein cattle population. J Dairy Sci. (2003) 86:360–8. 10.3168/jds.S0022-0302(03)73614-5 12613879

[14] Casas E Lunstra DD Stone RT. Quantitative trait loci for male reproductive traits in beef cattle. Anim Genet. (2004) 35:451–3. 10.1111/j.1365-2052.2004.01190.x 15566467

[15] Feugang JM Kaya A Page GP Chen L Mehta T Hirani K. Two-stage genome-wide association study identifies integrin beta 5 as having potential role in bull fertility. BMC Genomics. (2009) 10:176. 10.1186/1471-2164-10-176 19393042 PMC2684547

[16] Pausch H Flisikowski K Jung S Emmerling R Edel C Götz K-U. Genome-wide association study identifies two major loci affecting calving ease and growth-related traits in cattle. Genetics. (2011) 187:289–97. 10.1534/genetics.110.124057 21059885 PMC3018322

[17] Cole JB Wiggans GR Ma L Sonstegard TS Lawlor TJ Crooker BA. Genome-wide association analysis of thirty one production, health, reproduction and body conformation traits in contemporary US Holstein cows. BMC Genomics. (2011) 12:408. 10.1186/1471-2164-12-408 21831322 PMC3176260

[18] Blaschek M Kaya A Zwald N Memili E Kirkpatrick BW. A whole-genome association analysis of noncompensatory fertility in Holstein bulls. J Dairy Sci. (2011) 94:4695–9. 10.3168/jds.2010-3728 21854943

[19] McClure MC Morsci NS Schnabel RD Kim JW Yao P Rolf MM. A genome scan for quantitative trait loci influencing carcass, post-natal growth and reproductive traits in commercial Angus cattle. Anim Genet. (2010) 41:597–607. 10.1111/j.1365-2052.2010.02063.x 20477797

[20] Fortes MRS Reverter A Hawken RJ Bolormaa S Lehnert SA. Candidate genes associated with testicular development, sperm quality, and hormone levels of inhibin, luteinizing hormone, and insulin-like growth factor 1 in Brahman bulls. Biol Reprod. (2012) 87:51–8. 10.1095/biolreprod.112.101089 22811567

[21] Fortes MRS Lehnert SA Bolormaa S Reich C Fordyce G Corbet NJ. Finding genes for economically important traits: Brahman cattle puberty. Anim Prod Sci. (2012) 52:143–50. 10.1071/AN11165

[22] Peñagaricano F Weigel KA Khatib H. Genome-wide association study identifies candidate markers for bull fertility in Holstein dairy cattle. Anim Genet. (2012) 43:65–71. 10.1111/j.1365-2052.2012.02350.x 22742504

[23] Pausch H Venhoranta H Wurmser C Hakala K Iso-Touru T Sironen A. A frameshift mutation in ARMC3 is associated with a tail stump sperm defect in Swedish Red (Bos taurus) cattle. BMC Genet. (2016) 17:49. 10.1186/s12863-016-0356-7 26923438 PMC4770540

[24] Iso-Touru T Wurmser C Venhoranta H Hiltpold M Savolainen T Sironen A. A splice donor variant in CCDC189 is associated with asthenospermia in Nordic Red dairy cattle. BMC Genomics. (2019) 20:286. 10.1186/s12864-019-5628-y 30975085 PMC6460654

[25] Han Y Peñagaricano F. Unravelling the genomic architecture of bull fertility in Holstein cattle. BMC Genet. (2016) 17:143. 10.1186/s12863-016-0454-6 27842509 PMC5109745

[26] Tüttelmann F Krenková P Römer S Nestorovic AR Ljujic M Štambergová A. A common haplotype of protamine 1 and 2 genes is associated with higher sperm counts. Int J Androl. (2010) 33:e240–8. 10.1111/j.1365-2605.2009.01003.x 19863670

[27] Giesecke K Hamann H Stock KF Woehlke A Sieme H Distl O. Evaluation of SPATA1-associated markers for stallion fertility. Anim Genet. (2009) 40:359–65. 10.1111/j.1365-2052.2008.01844.x 19220231

[28] Pang Y-Y Lu RJ-H Chen P-Y. Behavioral epigenetics: perspectives based on experience-dependent epigenetic inheritance. Epigenomes. (2019) 3:18. 10.3390/epigenomes3030018 PMC8594690 34968228

[29] Seisenberger S Andrews S Krueger F Arand J Walter J Santos F. The dynamics of genome-wide DNA methylation reprogramming in mouse primordial germ cells. Mol Cell. (2012) 48:849–62. 10.1016/j.molcel.2012.11.001 23219530 PMC3533687

[30] Stewart KR Veselovska L Kelsey G. Establishment and functions of DNA methylation in the germline. Epigenomics. (2016) 8:1399–413. 10.2217/epi-2016-0056 27659720 PMC5066131

[31] Hammoud SS Low DHP Yi C Lee CL Oatley JM Payne CJ. Transcription and imprinting dynamics in developing postnatal male germline stem cells. Genes Dev. (2015) 29:2312–24. 10.1101/gad.261925.115 26545815 PMC4647563

[32] Hill PWS Leitch HG Requena CE Sun Z Amouroux R Roman-Trufero M. Epigenetic reprogramming enables the transition from primordial germ cell to gonocyte. Nature. (2018) 555:392–6. 10.1038/nature25964 29513657 PMC5856367

[33] Rawe VY Díaz ES Abdelmassih R Wójcik C Morales P Sutovsky P. The role of sperm proteasomes during sperm aster formation and early zygote development: implications for fertilization failure in humans. Hum Reprod. (2008) 23:573–80. 10.1093/humrep/dem385 18089554

[34] Tarnasky H Cheng M Ou Y Thundathil JC Oko R Van Der Hoorn FA. Gene trap mutation of murine Outer dense fiber protein-2 gene can result in sperm tail abnormalities in mice with high percentage chimaerism. BMC Dev Biol. (2010) 10:67. 10.1186/1471-213X-10-67 20550699 PMC2894780

[35] Zhao W Li Z Ping P Wang G Yuan X Sun F. Outer dense fibers stabilize the axoneme to maintain sperm motility. J Cell Mol Med. (2018) 22:1755–68. 10.1111/jcmm.13457 29168316 PMC5824370

[36] Zhao C Huo R Wang FQ Lin M Zhou ZM Sha JH. Identification of several proteins involved in regulation of sperm motility by proteomic analysis. Fertil Steril. (2007) 87:436–8. 10.1016/j.fertnstert.2006.06.057 17074334

[37] Ito J Parrington J Fissore RA. PLCζ and its role as a trigger of development in vertebrates. Mol Reprod Dev. (2011) 78:846–53. 10.1002/mrd.21359 21823187

[38] Aarabi M Balakier H Bashar S Moskovtsev SI Sutovsky P Librach CL. Sperm-derived WW domain-binding protein, PAWP, elicits calcium oscillations and oocyte activation in humans and mice. FASEB J. (2014) 28:4434–40. 10.1096/fj.14-256495 24970390

[39] Satouh Y Nozawa K Ikawa M. Sperm postacrosomal WW domain-binding protein is not required for mouse egg activation. Biol Reprod. (2015) 93:94. 10.1095/biolreprod.115.131441 26377222

[40] Velho A Wang H Koenig L Grant KE Menezes ES Kaya A. Expression dynamics of Integrin Subunit Beta 5 in bovine gametes and embryos imply functions in male fertility and early embryonic development. Andrologia. (2019) 51:e13305. 10.1111/and.13305 31090238

[41] Chang YM Gianola D Heringstad B Klemetsdal G. Effects of trait definition on genetic parameter estimates and sire evaluation for clinical mastitis with threshold models. Anim Sci. (2004) 79:355–63. 10.1017/S1357729800090226

[42] Park Y-J Kwon W-S Oh S-A Pang M-G. Fertility-related proteomic profiling bull spermatozoa separated by percoll. J Proteome Res. (2012) 11:4162–8. 10.1021/pr300248s 22794312

[43] Calven P Rolland AD Jégou B Pineau C. Testicular postgenomics: targeting the regulation of spermatogenesis. Philos Trans R Soc B Biol Sci. (2010) 365:1481–500. 10.1098/rstb.2009.0294 20403865 PMC2871924

[44] Kasimanickam RK Kasimanickam VR Arangasamy A Kastelic JP. Sperm and seminal plasma proteomics of high- versus low-fertility Holstein bulls. Theriogenology. (2019) 126:41–8. 10.1016/j.theriogenology.2018.11.032 30529997

[45] Sharma R Agarwal A Mohanty G Hamada AJ Gopalan B Willard B. Proteomic analysis of human spermatozoa proteins with oxidative stress. Reprod Biol Endocrinol. (2013) 11:48. 10.1186/1477-7827-11-48 23688036 PMC3716960

[46] Bastián Y Roa-Espitia AL Mújica A Hernández-González EO. Calpain modulates capacitation and acrosome reaction through cleavage of the spectrin cytoskeleton. Reproduction. (2010) 140:673–84. 10.1530/REP-09-0545 20716611

[47] Shisheva A Südhof TC Czech MP. Cloning, characterization, and expression of a novel GDP dissociation inhibitor isoform from skeletal muscle. Mol Cell Biol. (1994) 14:3459–68. 10.1128/MCB.14.5.3459 7513052 PMC358710

[48] Burska UL Harle VJ Coffey K Darby S Ramsey H O'Neill D. Deubiquitinating enzyme Usp12 is a novel co-activator of the androgen receptor. J Biol Chem. (2013) 288:32641–50. 10.1074/jbc.M113.485912 24056413 PMC3820899

[49] Orlowski M Reznik S Ayala J Pierotti AR. Endopeptidase 24.15 from rat testes. Isolation of the enzyme and its specificity toward synthetic and natural peptides, including enkephalin-containing peptides. Biochem J. (1989) 261:951–8. 10.1042/bj2610951 2803255 PMC1138921

[50] Sosa MA Barbieri MA Bertini F. Binding of β-galactosidase from rat epididymal fluid to the sperm surface by high-affinity sites different from phosphomannosyl receptors. Reproduction. (1991) 93:279–85. 10.1530/jrf.0.0930279 1787447

[51] Baumgart E Lenk SV Loening SA Jung K. Tissue inhibitors of metalloproteinases 1 and 2 in human seminal plasma and their association with spermatozoa. Int J Androl. (2002) 25:369–71. 10.1046/j.1365-2605.2002.00383.x 12406369

[52] Shimokawa KI Katayama M Matsuda Y Takahashi H Hara I Sato H. Matrix metalloproteinase (MMP)-2 and MMP-9 activities in human seminal plasma. Mol Hum Reprod. (2002) 8:32–6. 10.1093/molehr/8.1.32 11756567

[53] Sanz L Calvete JJ Mann K Schäfer W Schmid ER Amselgruber W. The complete primary structure of the spermadhesin AWN, a zona pellucida-binding protein isolated from boar spermatozoa. FEBS Lett. (1992) 300:213–8. 10.1016/0014-5793(92)80848-B 1555646

[54] Romero A Romao MJ Varela PF Kölln I Dias JM Carvalho AL. The crystal structures of two spermadhesins reveal the CUB domain fold. Nat Struct Biol. (1997) 4:783–8. 10.1038/nsb1097-783 9334740

[55] Solís D Romero A Jiménez M Díaz-Mauriño T Calvete JJ. Binding of mannose-6-phosphate and heparin by boar seminal plasma PSP-II, a member of the spermadhesin protein family. FEBS Lett. (1998) 431:273–8. 10.1016/S0014-5793(98)00772-8 9708918

[56] Dostálová Z Calvete JJ Sanz L Hettel C Riedel D Töpfer-Petersen E. Immunolocalization and Quantitation of Acidic Seminal Fluid Protein (aSFP) in ejaculated, swim-up, and capacitated bull spermatozoa. Biol Chem Hoppe Seyler. (1994) 375:457–62. 10.1515/bchm3.1994.375.7.457 7945995

[57] Moura AA Souza CE Stanley BA Chapman DA Killian GJ. Proteomics of cauda epididymal fluid from mature Holstein bulls. J Proteomics. (2010) 73:2006–20. 10.1016/j.jprot.2010.06.005 20601273

[58] Bustamante-Filho IC Salton GD Munari FM Schneider MR Mattos RC Laurino JP. Recombinant expression and purification of the bovine acidic seminal fluid protein. Anim Reprod. (2014) 11:96–103.

[59] Go KJ Wolf DP. Albumin-mediated changes in sperm sterol content during capacitation. Biol Reprod. (1985) 32:145–53. 10.1095/biolreprod32.1.145 3971008

[60] Visconti PE Kopf GS. Regulation of protein phosphorylation during sperm capacitation. Biol Reprod. (1998) 59:1–6. 10.1095/biolreprod59.1.1 9674985

[61] Atkinson DE. The energy charge of the adenylate pool as a regulatory parameter. Interaction with Feedback Modifiers. Biochemistry. (1968) 7:4030–4. 10.1021/bi00851a033 4972613

[62] Schoff PK Cheetham J Lardy HA. Adenylate kinase activity in ejaculated bovine sperm flagella. J Biol Chem. (1989) 264:6086–91. 10.1016/S0021-9258(18)83316-6 2539368

[63] Cao W Haig-Ladewig L Gerton GL Moss SB. Adenylate kinases 1 and 2 are part of the accessory structures in the mouse sperm flagellum. Biol Reprod. (2006) 75:492–500. 10.1095/biolreprod.106.053512 16790685

[64] Gibbons R Adeoya-Osiguwa SA Fraser LR. A mouse sperm decapacitation factor receptor is phosphatidylethanolamine-binding protein 1. Reproduction. (2005) 130:497–508. 10.1530/rep.1.00792 16183867

[65] Nixon B MacIntyre DA Mitchell LA Gibbs GM O'Bryan M Aitken RJ. The identification of mouse sperm-surface-associated proteins and characterization of their ability to act as decapacitation factors. Biol Reprod. (2006) 74:275–87. 10.1095/biolreprod.105.044644 16221991

[66] Desnoyers L Manjunath P. Major proteins of bovine seminal plasma exhibit novel interactions with phospholipid. J Biol Chem. (1992) 267:10149–55. 10.1016/S0021-9258(19)50212-5 1577785

[67] Manjunath P Lefebvre J Jois PS Fan J Wright MW. New nomenclature for mammalian BSP genes. Biol Reprod. (2009) 80:394–7. 10.1095/biolreprod.108.074088 18923155 PMC2844493

[68] D'Amours O Frenette G Fortier M Leclerc P Sullivan R. Proteomic comparison of detergent-extracted sperm proteins from bulls with different fertility indexes. Reproduction. (2010) 139:545–56. 10.1530/REP-09-0375 19952166

[69] Gwathmey TYM Ignotz GG Mueller JL Manjunath P Suarez SS. Bovine seminal plasma proteins PDC-109, BSP-A3, and BSP-30-kDa share functional roles in storing sperm in the oviduct. Biol Reprod. (2006) 75:501–7. 10.1095/biolreprod.106.053306 16790686

[70] Saalmann A Mnz S Ellerbrock K Ivell R Kirchhoff C. Novel sperm-binding proteins of epididymal origin contain four fibronectin type II-modules. Mol Reprod Dev. (2001) 58:88–100. 10.1002/1098-2795(200101)58:1<88::AID-MRD12>3.0.CO;2-D 11144225

[71] Glickman MH Ciechanover A. The ubiquitin-proteasome proteolytic pathway: destruction for the sake of construction. Physiol Rev. (2002) 82:373–428. 10.1152/physrev.00027.2001 11917093

[72] Morales P Kong M Pizarro E Pasten C. Participation of the sperm proteasome in human fertilization. Hum Reprod. (2003) 18:1010–7. 10.1093/humrep/deg111 12721178

[73] Gómez-Puertas P Martín-Benito J Carrascosa JL Willison KR Valpuesta JM. The substrate recognition mechanisms in chaperonins. J Mol Recognit. (2004) 17:85–94. 10.1002/jmr.654 15027029

[74] Souès S Kann ML Fouquet JP Melki R. The cytosolic chaperonin CCT associates to cytoplasmic microtubular structures during mammalian spermiogenesis and to heterochromatin in germline and somatic cells. Exp Cell Res. (2003) 288:363–73. 10.1016/S0014-4827(03)00248-9 12915127

[75] Zalata A El-Samanoudy AZ Shaalan D El-Baiomy Y Taymour M Mostafa T. Seminal clusterin gene expression associated with seminal variables in fertile and infertile men. J Urol. (2012) 188:1260–4. 10.1016/j.juro.2012.06.012 22902018

[76] O'bryan MK Murphy BF Liu DY Clarke GN Baker HWG. The use of anticlusterin monoclonal antibodies for the combined assesment of human sperm morphology and acrosome integrity. Hum Reprod. (1994) 9:1490–6. 10.1093/oxfordjournals.humrep.a138736 7989511

[77] Kimmins S Sassone-Corsi P. Chromatin remodelling and epigenetic features of germ cells. Nature. (2005) 434:583–9. 10.1038/nature03368 15800613

[78] Levin ER Gardner DG Samson WK. Natriuretic peptides. N Engl J Med. (1998) 339:321–8. 10.1056/NEJM199807303390507 9682046

[79] Öztop M Cinar K Turk S. Immunolocalization of natriuretic peptides and their receptors in goat (Capra hircus) heart. Biotech Histochem. (2018) 93:389–404. 10.1080/10520295.2018.1425911 30124338

[80] Sellitti DF Koles N Mendona MC. Regulation of C-type natriuretic peptide expression. Peptides. (2011) 32:1964–71. 10.1016/j.peptides.2011.07.013 21816187

[81] Chrisman TD Schulz S Potter LR Garbers DL. Seminal plasma factors that cause large elevations in cellular cyclic GMP are C-type natriuretic peptides. J Biol Chem. (1993) 268:3698–703. 10.1016/S0021-9258(18)53749-2 8094083

[82] Nielsen SJ Gøtze JP Jensen HL Rehfeld JF. ProCNP and CNP are expressed primarily in male genital organs. Regul Pept. (2008) 146:204–12. 10.1016/j.regpep.2007.09.022 17928074

[83] Özbek M Hitit M Öztop M Beyaz F Ergün E Ergün L. Spatiotemporal expression patterns of natriuretic peptides in rat testis and epididymis during postnatal development. Andrologia. (2019) 51: e13387. 10.1111/and.13387 31661170

[84] Xia H Chen Y Wu KJ Zhao H Xiong CL Huang DH. Role of C-type natriuretic peptide in the function of normal human sperm. Asian J Androl. (2016) 18:80–4. 10.4103/1008-682X.150254 25926602 PMC4736361

[85] Chang TSK Morton B. Epididymal sulfhydryl oxidase: a sperm-protective enzyme from the male reproductive tract. Biochem Biophys Res Commun. (1975) 66:309–15. 10.1016/S0006-291X(75)80329-9 1164427

[86] Ostrowski MC Kistler WS Williams-Ashman HG. A flavoprotein responsible for the intense sulfhydryl oxidase activity of rat seminal vesicle secretion. Biochem Biophys Res Commun. (1979) 87:171–6. 10.1016/0006-291X(79)91662-0 454397

[87] Cornwall GA Vindivich D Tillman S Chang TS. The effect of sulfhydryl oxidation on the morphology of immature hamster epididymal spermatozoa induced to acquire motility in vitro. Biol Reprod. (1988) 39:141–55. 10.1095/biolreprod39.1.141 3207793

[88] Tury A Mairet-Coello G Esnard-Fève A Benayoun B Risold PY Griffond B. Cell-specific localization of the sulphydryl oxidase QSOX in rat peripheral tissues. Cell Tissue Res. (2006) 323:91–103. 10.1007/s00441-005-0043-x 16160860

[89] Barondes SH Cooper DNW Gitt MA Leffler H. Galectins. Structure and function of a large family of animal lectins. J Biol Chem. (1994) 269:20807–10. 10.1016/S0021-9258(17)31891-4 8063692

[90] Thijssen VLJL Poirier F Baum LG Griffioen AW. Galectins in the tumor endothelium: opportunities for combined cancer therapy. Blood. (2007) 110:2819–27. 10.1182/blood-2007-03-077792 17591944

[91] Akahani S Nangia-Makker P Inohara H Kim H-RC Raz A. Galectin-3: a novel antiapoptotic molecule with a functional BH1 (NWGR) domain of Bcl-2 family. Cancer Res. (1997) 57:5272–6. 9393748

[92] Özbek M Hitit M Yildirim N Özgenç Ö Ergün E Ergün L. Expression pattern of galectin-1 and galectin-3 in rat testes and epididymis during postnatal development. Acta Histochem. (2018) 120:814–27. 10.1016/j.acthis.2018.09.006 30241677

[93] Kovak MR Saraswati S Schoen DJ Diekman AB. Investigation of galectin-3 function in the reproductive tract by identification of binding ligands in human seminal plasma. Am J Reprod Immunol. (2014) 72:403–12. 10.1111/aji.12273 24863808 PMC4180297

[94] Gomes FP Diedrich JK Saviola AJ Memili E Moura AA Yates JR III. EThcD and 213 nm UVPD for top-down analysis of bovine seminal plasma proteoforms on electrophoretic and chromatographic time frames. Anal Chem. (2020) 92:2979–87. 10.1021/acs.analchem.9b03856 31962043

[95] Krawetz SA. Paternal contribution: new insights and future challenges. Nat Rev Genet. (2005) 6:633–42. 10.1038/nrg1654 16136654

[96] Selvaraju S Parthipan S Somashekar L Kolte AP Binsila BK Arangasamy A. Occurrence and functional significance of the transcriptome in bovine (Bos taurus) spermatozoa. Sci Rep. (2017) 7:42392. 10.1038/srep42392 28276431 PMC5343582

[97] Bartel DP. MicroRNAs: genomics, biogenesis, mechanism, and function. Cell. (2004) 116:281–97. 10.1016/S0092-8674(04)00045-5 14744438

[98] Wang X Yang C Guo F Zhang Y Ju Z Jiang Q. Integrated analysis of mRNAs and long noncoding RNAs in the semen from Holstein bulls with high and low sperm motility. Sci Rep. (2019) 9:2092. 10.1038/s41598-018-38462-x 30765858 PMC6376035

[99] Rego JPA Martins JM Wolf CA Van Tilburg M Moreno F Monteiro-Moreira AC. Proteomic analysis of seminal plasma and sperm cells and their associations with semen freezability in Guzerat bulls. J Anim Sci. (2016) 94:5308–20. 10.2527/jas.2016-0811 28046165

[100] Delbridge ML Lingenfelter PA Disteche CM Graves JAM. The candidate spermatogenesis gene RBMY has a homologue on the human X chromosome. Nat Genet. (1999) 22:223–4. 10.1038/10279 10391206

[101] Yeste M Morató R Rodríguez-Gil JE Bonet S Prieto-Martínez N. Aquaporins in the male reproductive tract and sperm: functional implications and cryobiology. Reprod Domest Anim. (2017) 52:12–27. 10.1111/rda.13082 29052330

[102] Card CJ Anderson EJ Zamberlan S Krieger KE Kaproth M Sartini BL. Cryopreserved bovine spermatozoal transcript profile as revealed by high-throughput ribonucleic acid sequencing. Biol Reprod. (2013) 88:41–9. 10.1095/biolreprod.112.103788 23303677

[103] Feugang JM Rodriguez-Osorio N Kaya A Wang H Page G Ostermeier GC. Transcriptome analysis of bull spermatozoa: implications for male fertility. Reprod Biomed Online. (2010) 21:312–24. 10.1016/j.rbmo.2010.06.022 20638337

[104] Légaré C Akintayo A Blondin P Calvo E Sullivan R. Impact of male fertility status on the transcriptome of the bovine epididymis. MHR Basic Sci Reprod Med. (2017) 23:355–69. 10.1093/molehr/gax019 28379507

[105] Stowe HM Calcatera SM Dimmick MA Andrae JG Duckett SK Pratt SL. The bull sperm microRNAome and the effect of fescue toxicosis on sperm microRNA expression. PLoS ONE. (2014) 9:e113163. 10.1371/journal.pone.0113163 25462855 PMC4251976

[106] Nixon B Stanger SJ Mihalas BP Reilly JN Anderson AL Tyagi S. The microRNA signature of mouse spermatozoa is substantially modified during epididymal maturation. Biol Reprod. (2015) 93:91. 10.1095/biolreprod.115.132209 26333995

[107] Capra E Turri F Lazzari B Cremonesi P Gliozzi TM Fojadelli I. Small RNA sequencing of cryopreserved semen from single bull revealed altered miRNAs and piRNAs expression between High-and Low-motile sperm populations. BMC Genomics. (2017) 18:14. 10.1186/s12864-016-3394-7 28052756 PMC5209821

[108] Fagerlind M Stålhammar H Olsson B Klinga-Levan K. Expression of mi RNA s in bull spermatozoa correlates with fertility rates. Reprod Domest Anim. (2015) 50:587–94. 10.1111/rda.12531 25998690

[109] Tscherner A Gilchrist G Smith N Blondin P Gillis D LaMarre J. MicroRNA-34 family expression in bovine gametes and preimplantation embryos. Reprod Biol Endocrinol. (2014) 12:85. 10.1186/1477-7827-12-85 25179211 PMC4162940

[110] Yuan S Tang C Zhang Y Wu J Bao J Zheng H. mir-34b/c and mir-449a/b/c are required for spermatogenesis, but not for the first cleavage division in mice. Biol Open. (2015) 4:212–23. 10.1242/bio.201410959 25617420 PMC4365490

[111] Liu W-M Pang RTK Chiu PCN Wong BPC Lao K Lee K-F. Sperm-borne microRNA-34c is required for the first cleavage division in mouse. Proc Natl Acad Sci. (2012) 109:490–4. 10.1073/pnas.1110368109 22203953 PMC3258645

[112] Zhu Z Li C Yang S Tian R Wang J Yuan Q. Dynamics of the transcriptome during human spermatogenesis: predicting the potential key genes regulating male gametes generation. Sci Rep. (2016) 6:19069. 10.1038/srep19069 26753906 PMC4750114

[113] Menezes ESB Badial PR El Debaky H Husna AU Ugur MR Kaya A. Sperm miR-15a and miR-29b are associated with bull fertility. Andrologia. (2020) 52:e13412. 10.1111/and.13412 31671225

[114] Zhang L Lu H Xin D Cheng H Zhou R. A novel ncRNA gene from mouse chromosome 5 trans-splices with Dmrt1 on chromosome 19. Biochem Biophys Res Commun. (2010) 400:696–700. 10.1016/j.bbrc.2010.08.130 20816665

[115] Ni M-J Hu Z-H Liu Q Liu M-F Lu M Zhang J-S. Identification and characterization of a novel non-coding RNA involved in sperm maturation. PLoS ONE. (2011) 6:e26053. 10.1371/journal.pone.0026053 22022505 PMC3192136

[116] Arun G Akhade VS Donakonda S Rao MRS. mrhl RNA, a long noncoding RNA, negatively regulates Wnt signaling through its protein partner Ddx5/p68 in mouse spermatogonial cells. Mol Cell Biol. (2012) 32:3140–52. 10.1128/MCB.00006-12 22665494 PMC3434522

[117] Anguera MC Ma W Clift D Namekawa S Kelleher RJ III Lee JT. Tsx produces a long noncoding RNA and has general functions in the germline, stem cells, and brain. PLoS Genet. (2011) 7:e1002248. 10.1371/journal.pgen.1002248 21912526 PMC3164691

[118] Fukusaki E. Application of metabolomics for high resolution phenotype analysis. Mass Spectrom. (2014) 3:S0045. 10.5702/massspectrometry.S0045 26819889 PMC4541153

[119] Guijas C Montenegro-Burke JR Warth B Spilker ME Siuzdak G. Metabolomics activity screening for identifying metabolites that modulate phenotype. Nat Biotechnol. (2018) 36:316–20. 10.1038/nbt.4101 29621222 PMC5937131

[120] Gromski PS Muhamadali H Ellis DI Xu Y Correa E Turner ML. A tutorial review: metabolomics and partial least squares-discriminant analysis–a marriage of convenience or a shotgun wedding. Anal Chim Acta. (2015) 879:10–23. 10.1016/j.aca.2015.02.012 26002472

[121] Dipresa S De Toni L Garolla A. New markers for predicting fertility of the male gametes in the post genomic era. Protein Pept Lett. (2018) 25:434–9. 10.2174/0929866525666180418120635 29667547

[122] Odet F Gabel S London RE Goldberg E Eddy EM. Glycolysis and mitochondrial respiration in mouse LDHC-null sperm. Biol Reprod. (2013) 88:91–5. 10.1095/biolreprod.113.108530 23486916 PMC4013879

[123] Chen X Hu C Dai J Chen L. Metabolomics analysis of seminal plasma in infertile males with kidney-yang deficiency: a preliminary study. Evid Based Complement Altern Med. (2015) 2015:892930. 10.1155/2015/892930 25945117 PMC4405216

[124] Jayaraman V Ghosh S Sengupta A Srivastava S Sonawat HM Narayan PK. Identification of biochemical differences between different forms of male infertility by nuclear magnetic resonance (NMR) spectroscopy. J Assist Reprod Genet. (2014) 31:1195–204. 10.1007/s10815-014-0282-4 24965760 PMC4156941

[125] Engel KM Baumann S Rolle-Kampczyk U Schiller J von Bergen M Grunewald S. Metabolomic profiling reveals correlations between spermiogram parameters and the metabolites present in human spermatozoa and seminal plasma. PLoS ONE. (2019) 14:e0211679. 10.1371/journal.pone.0211679 30785892 PMC6382115

[126] Menezes EB Velho ALC Santos F Dinh T Kaya A Topper E. Uncovering sperm metabolome to discover biomarkers for bull fertility. BMC Genomics. (2019) 20:714. 10.1186/s12864-019-6074-6 31533629 PMC6749656

[127] Memili E Moura AA Kaya A. Metabolomes of sperm and seminal plasma associated with bull fertility. Anim Reprod Sci. (2020) 220:106355. 10.1016/j.anireprosci.2020.106355 32273206

[128] Velho ALC Menezes E Dinh T Kaya A Topper E Moura AA. Metabolomic markers of fertility in bull seminal plasma. PLoS ONE. (2018) 13:e0195279. 10.1371/journal.pone.0195279 29634739 PMC5892889

[129] Mann T. Studies on the metabolism of semen3. Fructose as a normal constituent of seminal plasma. Site of formation and function of fructose in semen. Biochem J. (1946) 40:481–91. 20273629 10.1042/bj0400481

[130] Baronos S. Seminal carbohydrate in boar and stallion. Reproduction. (1971) 24:303–5. 10.1530/jrf.0.0240303 5551420

[131] Mendoza G White IG Chow P. Studies of chemical components of Angora goat seminal plasma. Theriogenology. (1989) 32:455–66. 10.1016/0093-691X(89)90012-5 16726692

[132] Matsuoka T Imai H Asakuma S Kohno H Fukui Y. Changes of fructose concentrations in seminal plasma and glucose and testosterone concentrations in blood plasma in rams over the course of a year. J Reprod Dev. (2006) 52:805–10. 10.1262/jrd.18039 16988491

[133] Kamp G Lauterwein J. Multinuclear magnetic resonance studies of boar seminal plasma. Biochim Biophys Acta. (1995) 1243:101–9. 10.1016/0304-4165(94)00117-G 7827097

[134] Sørensen MB Bergdahl IA Hjøllund NHI Bonde JPE Stoltenberg M Ernst E. Zinc, magnesium and calcium in human seminal fluid: relations to other semen parameters and fertility. Mol Hum Reprod. (1999) 5:331–7. 10.1093/molehr/5.4.331 10321804

[135] Yi Y-J Sutovsky M Kennedy C Sutovsky P. Identification of the inorganic pyrophosphate metabolizing, ATP substituting pathway in mammalian spermatozoa. PLoS ONE. (2012) 7:e34524. 10.1371/journal.pone.0034524 22485177 PMC3317647

[136] Newairy A-SA Salama AF Hussien HM Yousef MI. Propolis alleviates aluminium-induced lipid peroxidation and biochemical parameters in male rats. Food Chem Toxicol. (2009) 47:1093–8. 10.1016/j.fct.2009.01.032 19425229

[137] Soggiu A Piras C Hussein HA De Canio M Gaviraghi A Galli A. Unravelling the bull fertility proteome. Mol Biosyst. (2013) 9:1188–95. 10.1039/c3mb25494a 23392320

[138] Ugur MR Dinh T Hitit M Kaya A Topper E Didion B. Amino acids of seminal plasma associated with freezability of bull sperm. Front cell Dev Biol. (2020) 7:347. 10.3389/fcell.2019.00347 31993417 PMC6970951

[139] Zamudio N Barau J Teissandier A Walter M Borsos M Servant N Bourc'his D. DNA methylation restrains transposons from adopting a chromatin signature permissive for meiotic recombination. Genes Dev. (2015) 29:1256–70. 10.1101/gad.257840.114 26109049 PMC4495397

[140] Miller D Brinkworth M Iles D. Paternal DNA packaging in spermatozoa: more than the sum of its parts? DNA, histones, protamines and epigenetics. Reproduction. (2010) 139:287–301. 10.1530/REP-09-0281 19759174

[141] Larson EL Vanderpool D Keeble S Zhou M Sarver BAJ Smith AD. Contrasting levels of molecular evolution on the mouse X chromosome. Genetics. (2016) 203:1841–57. 10.1534/genetics.116.186825 27317678 PMC4981281

[142] Allegrucci C Thurston A Lucas E Young L. Epigenetics and the germline. Reproduction. (2005) 129:137–49. 10.1530/rep.1.00360 15695608

[143] Yoder JA Soman NS Verdine GL Bestor TH. DNA (cytosine-5)-methyltransferases in mouse cells and tissues. Studies with a mechanism-based probe. J Mol Biol. (1997) 270:385–95. 10.1006/jmbi.1997.1125 9237905

[144] Jang HS Shin WJ Lee JE Do JT. CpG and non-CpG methylation in epigenetic gene regulation and brain function. Genes (Basel). (2017) 8:148. 10.3390/genes8060148 28545252 PMC5485512

[145] Kato Y Kaneda M Hata K Kumaki K Hisano M Kohara Y. Role of the Dnmt3 family in de novo methylation of imprinted and repetitive sequences during male germ cell development in the mouse. Hum Mol Genet. (2007) 16:2272–80. 10.1093/hmg/ddm179 17616512

[146] Seisenberger S Peat JR Hore TA Santos F Dean W Reik W. Reprogramming DNA methylation in the mammalian life cycle: building and breaking epigenetic barriers. Philos Trans R Soc B Biol Sci. (2013) 368:20110330. 10.1098/rstb.2011.0330 23166394 PMC3539359

[147] Kriaucionis S Heintz N. The nuclear DNA base 5-hydroxymethylcytosine is present in Purkinje neurons and the brain. Science. (2009) 324:929–30. 10.1126/science.1169786 19372393 PMC3263819

[148] Tahiliani M Koh KP Shen Y Pastor WA Bandukwala H Brudno Y. Conversion of 5-methylcytosine to 5-hydroxymethylcytosine in mammalian DNA by MLL partner TET1. Science. (2009) 324:930–35. 10.1126/science.1170116 19372391 PMC2715015

[149] Ito S Shen L Dai Q Wu SC Collins LB Swenberg JA. Tet proteins can convert 5-methylcytosine to 5-formylcytosine and 5-carboxylcytosine. Science. (2011) 333:1300–3. 10.1126/science.1210597 21778364 PMC3495246

[150] Wu H Zhang Y. Reversing DNA methylation: mechanisms, genomics, and biological functions. Cell. (2014) 156:45–68. 10.1016/j.cell.2013.12.019 24439369 PMC3938284

[151] Neri F Incarnato D Krepelova A Parlato C Oliviero S. Methylation-assisted bisulfite sequencing to simultaneously map 5fC and 5caC on a genome-wide scale for DNA demethylation analysis. Nat Protoc. (2016) 11:1191–205. 10.1038/nprot.2016.063 27281647

[152] Moore LD Le T Fan G. DNA methylation and its basic function. Neuropsychopharmacology. (2013) 38:23–38. 10.1038/npp.2012.112 22781841 PMC3521964

[153] Donkin I Barrès R. Sperm epigenetics and influence of environmental factors. Mol Metab. (2018) 14:1–11. 10.1016/j.molmet.2018.02.006 29525406 PMC6034033

[154] Kiefer H Perrier J-P. DNA methylation in bull spermatozoa: evolutionary impacts, interindividual variability, and contribution to the embryo. Can J Anim Sci. (2019) 100:1–16. 10.1139/cjas-2019-0071

[155] Liu S Fang L Zhou Y Santos DJA Xiang R Daetwyler HD. Analyses of inter-individual variations of sperm DNA methylation and their potential implications in cattle. BMC Genomics. (2019) 20:888. 10.1186/s12864-019-6228-6 31752687 PMC6873545

[156] Naz RK Joseph A Lee Y Ahmad K Bhargava MM. Expression of scatter factor/hepatocyte growth factor is regionally correlated with the initiation of sperm motility in murine male genital tract: is scatter factor/hepatocyte growth factor involved in initiation of sperm motility? Mol Reprod Dev. (1994) 38:431–9. 10.1002/mrd.1080380411 7980952

[157] Herness EA Naz RK. Presence and tyrosine phosphorylation of c-met receptor in human sperm. J Androl. (1999) 20:640–7. 10520577

[158] Fang L Zhou Y Liu S Jiang J Bickhart DM Null DJ. Comparative analyses of sperm DNA methylomes among human, mouse and cattle provide insights into epigenomic evolution and complex traits. Epigenetics. (2019) 14:260–76. 10.1080/15592294.2019.1582217 30810461 PMC6557555

[159] Zhou Y Connor EE Bickhart DM Li C Baldwin RL Schroeder SG. Comparative whole genome DNA methylation profiling of cattle sperm and somatic tissues reveals striking hypomethylated patterns in sperm. Gigascience. (2018) 7:giy039. 10.1093/gigascience/giy039 29635292 PMC5928411

[160] Ma D-D Wang D-H Yang W-X. Kinesins in spermatogenesis. Biol Reprod. (2017) 96:267–76. 10.1095/biolreprod.116.144113 28203733

[161] Rathke C Baarends WM Awe S Renkawitz-Pohl R. Chromatin dynamics during spermiogenesis. Biochim Biophys Acta. (2014) 1839:155–68. 10.1016/j.bbagrm.2013.08.004 24091090

[162] Govin J Caron C Lestrat C Rousseaux S Khochbin S. The role of histones in chromatin remodelling during mammalian spermiogenesis. Eur J Biochem. (2004) 271:3459–69. 10.1111/j.1432-1033.2004.04266.x 15317581

[163] de Oliveira R V Dogan S Belser LE Kaya A Topper E Moura A. Molecular morphology and function of bull spermatozoa linked to histones and associated with fertility. Reproduction. (2013) 146:263–72. 10.1530/REP-12-0399 23904564

[164] Kutchy NA Velho A Menezes ESB Jacobsen M Thibaudeau G Wills RW. Testis specific histone 2B is associated with sperm chromatin dynamics and bull fertility-a pilot study. Reprod Biol Endocrinol. (2017) 15:59. 10.1186/s12958-017-0274-1 28764714 PMC5539985

[165] Dogan S Vargovic P Oliveira R Belser LE Kaya A Moura A. Sperm protamine-status correlates to the fertility of breeding bulls. Biol Reprod. (2015) 92:91–2. 10.1095/biolreprod.114.124255 25673563

[166] Kutchy NA Menezes ESB Ugur MR Husna AU ElDebaky H Evans HC. Sperm cellular and nuclear dynamics associated with bull fertility. Anim Reprod Sci. (2019) 211:106203. 10.1016/j.anireprosci.2019.106203 31785643

[167] Kutchy NA Menezes ESB Chiappetta A Tan W Wills RW Kaya A. Acetylation and methylation of sperm histone 3 lysine 27 (H3K27ac and H3K27me3) are associated with bull fertility. Andrologia. (2018) 50:e12915. 10.1111/and.12915 29057498

[168] Verma A Rajput S Kumar S De S Chakravarty AK Kumar R. Differential histone modification status of spermatozoa in relation to fertility of buffalo bulls. J Cell Biochem. (2015) 116:743–53. 10.1002/jcb.25029 25501625

[169] Underhill DA. Genetic and biochemical diversity in the Pax gene family. Biochem Cell Biol. (2000) 78:629–38. 10.1139/bcb-78-5-629 11103953

[170] Kamachi Y Uchikawa M Kondoh H. Pairing SOX off: with partners in the regulation of embryonic development. Trends Genet. (2000) 16:182–7. 10.1016/S0168-9525(99)01955-1 10729834

[171] Owens JC Detweiler CS Li JJ. CDC45 is required in conjunction with CDC7/DBF4 to trigger the initiation of DNA replication. Proc Natl Acad Sci. (1997) 94:12521–6. 10.1073/pnas.94.23.12521 9356482 PMC25024

[172] Slotman JA Paul MW Carofiglio F de Gruiter HM Vergroesen T Koornneef L. Super-resolution imaging of RAD51 and DMC1 in DNA repair foci reveals dynamic distribution patterns in meiotic prophase. PLoS Genet. (2020) 16:e1008595. 10.1371/journal.pgen.1008595 32502153 PMC7310863

[173] Sancar A. Excision repair invades the territory of mismatch repair. Nat Genet. (1999) 21:247–9. 10.1038/6753 10080168

[174] Ji G Long Y Zhou Y Huang C Gu A Wang X. Common variants in mismatch repair genes associated with increased risk of sperm DNA damage and male infertility. BMC Med. (2012) 10:49. 10.1186/1741-7015-10-49 22594646 PMC3378460

[175] Nakaki F Hayashi K Ohta H Kurimoto K Yabuta Y Saitou M. Induction of mouse germ-cell fate by transcription factors in vitro. Nature. (2013) 501:222–6. 10.1038/nature12417 23913270

[176] Seki Y. PRDM14 is a unique epigenetic regulator stabilizing transcriptional networks for pluripotency. Front Cell Dev Biol. (2018) 6:12. 10.3389/fcell.2018.00012 29487849 PMC5816753

[177] Okashita N Kumaki Y Ebi K Nishi M Okamoto Y Nakayama M. PRDM14 promotes active DNA demethylation through the ten-eleven translocation (TET)-mediated base excision repair pathway in embryonic stem cells. Development. (2014) 141:269–80. 10.1242/dev.099622 24335252

[178] Grabole N Tischler J Hackett JA Kim S Tang F Leitch HG. Prdm14 promotes germline fate and naive pluripotency by repressing FGF signalling and DNA methylation. EMBO Rep. (2013) 14:629–37. 10.1038/embor.2013.67 23670199 PMC3701237

[179] Mallol A Guirola M Payer B. PRDM14 controls X-chromosomal and global epigenetic reprogramming of H3K27me3 in migrating mouse primordial germ cells. Epigenetics Chromatin. (2019) 12:38. 10.1186/s13072-019-0284-7 31221220 PMC6585054

[180] Yamaji M Seki Y Kurimoto K Yabuta Y Yuasa M Shigeta M. Critical function of Prdm14 for the establishment of the germ cell lineage in mice. Nat Genet. (2008) 40:1016. 10.1038/ng.186 18622394

[181] Yamaji M Ueda J Hayashi K Ohta H Yabuta Y Kurimoto K. PRDM14 ensures naive pluripotency through dual regulation of signaling and epigenetic pathways in mouse embryonic stem cells. Cell Stem Cell. (2013) 12:368–82. 10.1016/j.stem.2012.12.012 23333148

[182] Zhang X Rice K Wang Y Chen W Zhong Y Nakayama Y. Maternally expressed gene 3 (MEG3) noncoding ribonucleic acid: isoform structure, expression, and functions. Endocrinology. (2010) 151:939–47. 10.1210/en.2009-0657 20032057 PMC2840681

[183] Hammoud SS Nix DA Zhang H Purwar J Carrell DT Cairns BR. Distinctive chromatin in human sperm packages genes for embryo development. Nature. (2009) 460:473–8. 10.1038/nature08162 19525931 PMC2858064

[184] Bernstein BE Mikkelsen TS Xie X Kamal M Huebert DJ Cuff J. A bivalent chromatin structure marks key developmental genes in embryonic stem cells. Cell. (2006) 125:315–26. 10.1016/j.cell.2006.02.041 16630819

[185] Zindy F den Besten W Chen B Rehg JE Latres E Barbacid M Pollard JW Sherr CJ Cohen PE Roussel MF. Control of spermatogenesis in mice by the cyclin D-dependent kinase inhibitors p18(Ink4c) and p19(Ink4d). Mol Cell Biol. (2001) 21:3244–55. 10.1128/MCB.21.9.3244-3255.2001 11287627 PMC86968

